# A Viral Nuclear Noncoding RNA Binds Re-localized Poly(A) Binding Protein and Is Required for Late KSHV Gene Expression

**DOI:** 10.1371/journal.ppat.1002300

**Published:** 2011-10-13

**Authors:** Sumit Borah, Nicole Darricarrère, Alicia Darnell, Jinjong Myoung, Joan A. Steitz

**Affiliations:** 1 Department of Molecular Biophysics and Biochemistry, Howard Hughes Medical Institute, Yale University, New Haven, Connecticut, United States of America; 2 Department of Cell Biology, Yale University School of Medicine, New Haven, Connecticut, United States of America; 3 Department of Microbiology and Immunology, Howard Hughes Medical Institute, University of California, San Francisco, California, United States of America; University of North Carolina at Chapel Hill, United States of America

## Abstract

During the lytic phase of infection, the gamma herpesvirus Kaposi's Sarcoma-Associated Herpesvirus (KSHV) expresses a highly abundant, 1.1 kb nuclear noncoding RNA of unknown function. We observe that this polyadenylated nuclear (PAN) RNA avidly binds host poly(A)-binding protein C1 (PABPC1), which normally functions in the cytoplasm to bind the poly(A) tails of mRNAs, regulating mRNA stability and translation efficiency. During the lytic phase of KSHV infection, PABPC1 is re-localized to the nucleus as a consequence of expression of the viral shutoff exonuclease (SOX) protein; SOX also mediates the host shutoff effect in which host mRNAs are downregulated while viral mRNAs are selectively expressed. We show that whereas PAN RNA is not required for the host shutoff effect or for PABPC1 re-localization, SOX strongly upregulates the levels of PAN RNA in transient transfection experiments. This upregulation is destroyed by the same SOX mutation that ablates the host shutoff effect and PABPC1 nuclear re-localization or by removal of the poly(A) tail of PAN. In cells induced into the KSHV lytic phase, depletion of PAN RNA using RNase H-targeting antisense oligonucleotides reveals that it is necessary for the production of late viral proteins from mRNAs that are themselves polyadenylated. Our results add to the repertoire of functions ascribed to long noncoding RNAs and suggest a mechanism of action for nuclear noncoding RNAs in gamma herpesvirus infection.

## Author Summary

Almost all eukaryotic messenger RNAs (mRNAs) have a string of 150–200 adenylates at the 3′ end. This poly(A) tail has been implicated as important for regulating mRNA translation, stability and export. During the lytic phase of infection of Kaposi's Sarcoma-Associated Herpesvirus (KSHV), a noncoding viral RNA is synthesized that resembles an mRNA in that it is transcribed by RNA polymerase II, is methyl-G capped at the 5′ end, and is polyadenylated at the 3′ end; yet this RNA is never exported to the cytoplasm for translation. Rather, it builds up in the nucleus to exceedingly high levels. We present evidence that the function of this abundant, polyadenylated nuclear (PAN) RNA is to bind poly(A) binding protein, which normally binds poly(A) tails of mRNAs in the cytoplasm but is re-localized into the nucleus during lytic KSHV infection. The interaction between PAN RNA and re-localized poly(A) binding protein is important for formation of new virus, in particular for the synthesis of proteins made late in infection. Our study provides new insight into the function of this noncoding RNA during KSHV infection and expands recent discoveries regarding re-localization of poly(A) binding protein during many viral infections.

## Introduction

Kaposi's Sarcoma-Associated Herpesvirus (KSHV) is the causative agent of several human cancers and immunoproliferative disorders, including Kaposi's Sarcoma, Multicentric Castleman's Disease and Primary Effusion Lymphoma [1], [2]. Like other herpesviruses, KSHV infection is characterized by two states: viral latency and lytic growth. During latency, very few viral genes are expressed, reducing the number of viral epitopes available to trigger a host immune response. Given appropriate but incompletely understood stimuli, the virus activates the lytic program of infection. This is characterized by three ordered waves of viral gene expression producing “immediate early,” “delayed early” and “late” proteins, as well as replication of the viral genome. Ultimately, the new genomes are packaged into virions, which are released from the cell for expansive host infection.

Upon KSHV entry into the lytic phase, an intronless viral noncoding (nc)RNA called polyadenylated nuclear (PAN) RNA, also known as T1.1 or nut-1, begins to be synthesized at unusually high levels [3], [4]. Although the 1.1 kb PAN RNA resembles an mRNA in being transcribed by RNA polymerase II, methyl-G capped at its 5′ end, and polyadenylated at its 3′ end, it is not exported to the cytoplasm for translation as are other viral transcripts. Instead, PAN RNA accumulates to astonishingly high levels, reaching ∼500,000 copies per nucleus and ultimately accounting for up to 80% of the total polyadenylated RNA in the cell [3]. Much has been learned regarding the mechanism that enables PAN RNA to accumulate to such high levels. Specifically, a 79-nucleotide element located near the 3′ end of the RNA, termed the expression and nuclear retention element (ENE), serves to stabilize the RNA in the nucleus [5], [6], [7]. Deletion of the ENE dramatically reduces the levels of transfected PAN RNA in HEK 293 cells, while insertion of the ENE into an intronless β-globin transcript significantly increases its nuclear levels. Insertion of the ENE has also been shown to enhance the abundance of nuclear pri-miRNAs [8]. It was hypothesized that a U-rich internal loop within the ENE engages the poly(A) tail, thereby sequestering it from deadenylases that initiate RNA decay [6], [7]. A recent x-ray crystal structure of the ENE complexed with oligo(A) reveals the formation of a triple helix that clamps the oligo(A) [9].

To address how PAN RNA contributes to lytic infection of KSHV, we began by investigating protein components of the PAN RNP and identified poly(A)-binding protein C1 (PABPC1). PABPC1 normally functions in the cytoplasm where it binds the poly(A) tails of mRNAs, regulating their stability by either antagonizing or enhancing the activity of cytoplasmic deadenylases [10], [11], [12], [13], [14]. PABPC1 also mediates circularization and enhances translation of mRNA via physical interactions with the translation initiation factor eIF4G, and assists in the export of mRNAs from the nucleus to the cytoplasm [14], [15], [16], [17]. However, since PAN RNA resides exclusively in the nucleus of KSHV-infected cells and does not shuttle (Conrad and Steitz, unpublished observations), re-localization of PABPC1 to the nucleus is a prerequisite for significant binding of PABPC1 to PAN RNA. Indeed, several groups have reported that re-localization of PABPC1 from the cytoplasm into the nucleus occurs during lytic KSHV infection of TIME endothelial cells and BC3 and BCBL1 TReX-RTA lymphoid cells [18], [19], [20]. The phenomenon is driven by the shutoff exonuclease (SOX) protein, as transient transfection of SOX into uninfected cells causes nuclear accumulation of PABPC1 in the absence of any other viral gene product [19]. SOX is also responsible for the host shutoff effect of the virus, which selectively downregulates host mRNAs while viral mRNAs persist [21], [22], [23]. SOX-mediated PABPC1 re-localization to the nucleus is critical for the host shutoff effect since knockdown of PABPC1 diminishes the ability of SOX to effect shutoff. GFP mRNA levels were downregulated in cells transfected with SOX, but less so in cells pre-treated with anti-PABPC1 siRNAs [19]. Furthermore, overexpression of PABPC1 targeted into the nucleus, using a nuclear retention signal from hnRNPC1, recapitulates aspects of host shutoff in the absence of any viral gene product [24]. Finally, a point mutation that disrupts the ability of SOX to re-localize PABPC1, but does not disrupt SOX's unrelated function as an alkaline DNase, ablates its ability to mediate host shutoff [19].

Here, we demonstrate that PAN RNA interacts with PABPC1 after it has re-localized to the nucleus during the lytic phase of KSHV infection. In transient transfection experiments, SOX strongly upregulates the levels of PAN RNA by a mechanism that is dependent on the ability of SOX to mediate the host shutoff effect, on the re-localization of PABPC1 into the nucleus and on the existence of a poly(A) tail on PAN RNA. In infected cultured cells activated into lytic phase, PAN RNA expression is coincident with the host shutoff effect, and correlates with PABPC1 re-localization from the cytoplasm into the nucleus. Yet, knockdown of PAN RNA using RNase H-targeting oligonucleotides shows that it is not required for the shutoff effect or the nuclear re-localization of PABPC1. Instead, the amount of virus released into the supernatant of cultured cells is severely reduced upon knockdown. This striking effect on viral titer is explained by our observation that knockdown of PAN RNA adversely affects the expression of a subset of viral genes during the late stage of lytic infection.

## Materials and Methods

### Cell lines, antibodies and transfection protocols

BCBL1 TReX-vector and TReX-RTA cells (gift from Jae Jung, USC) were maintained in RPMI supplemented with pen/strep, L-glutamine and 20% tetracycline-compatible FBS (Clonetech). iSLK.219, 293 DC-SIGN (gift from Robert Means and Sabine Lang, Yale University) and 293T cells were maintained in DMEM supplemented with pen/strep, L-glutamine and 10% FBS. 293 tet-on cells were maintained in DMEM supplemented with pen/strep, L-glutamine and 10% tetracycline-compatible FBS. Anti-orf6/SSB was a gift from G. Hayward (Johns Hopkins University), anti-LANA1 and anti-orf4 were gifts from Y. Chang (University of Pittsburg), anti-K8.1 antibody was from Advanced Biochemicals Incorporated, anti-orf65 antibody was a gift from G. Miller (Yale University), anti-myc antibody was from Santa Cruz Biotechnology. Electroporation was conducted in 0.4 cm electroporation cuvettes at 975 µF/220 mV for BCBL1 cells, and 975 µF/210 mV for iSLK.219 cells. 293T cells were transfected with MirusTransIT reagent according to the manufacturer's protocol. For some oligonucleotide knockdown experiments in BCBL1 TReX-RTA cells, cultures were first synchronized into S phase by serum starvation for 24 hours, followed by the re-addition of serum and incubation for 16 hours [25], [26]. Cells were then electroporated with RNase H-targeting modified oligonucleotides and allowed to recuperate overnight in RPMI supplemented with 20% tetracycline-compatible FBS (Clonetech), followed by induction the next day with 0.2 µg/mL doxycycline. Other transfection methods were tested, such as nucleofection with both Amaxa and Mirus reagents. Nucleofection with Amaxa reagent, as per the manufacturer's suggestions for BCBL1 cells, gave results comparable in terms of knockdown efficiency and cell death to the electroporation method used here.

### Purification of PAN RNP

KSHV-infected BCBL1 TReX-vector or TReX-RTA cultures were grown to a density of 0.8 million cells/mL, and then treated with 2 ug/mL doxycycline for 24 hours. 100 million cells were pelleted, washed with PBS, resuspended in 500 uL 50 mM Tris-HCl, pH 7, 100 mM KCl, 10 mM MgCl_2_, 1 mM DTT, 10% glycerol, 1 mM ATP, protease inhibitor cocktail (CalbioChem) and RNase Inhibitor (Roche), and syringe lysed by 10 passages through a 20G needle, 15 passages through a 25G7/8 needle and 20 passages through a 27G needle. Lysate was clarified by spinning at 14,000 x g for 10 minutes at 4°C, before nutating with 500 µL bed volume DEAE-sepharose resin that had been pre-equilibriated with lysis buffer. After 4 hours at 4°C, the resin was pelleted at 3000 rpm for 1 minute at 4°C, washed twice with lysis buffer, and subjected to successive batch elutions with increasing concentrations of KCl in lysis buffer. Maximal yield of the PAN RNP was observed in the 0.3 M KCl fraction. 1 mM MgCl_2_, 1.5 mM ATP, 5 mM creatine phosphate and 2–5 pmol/µL of biotinylated 2′-*O*-methylated anti-PAN oligonucleotides, complementary to nucleotides 70 to 89 and 993 to 1012 of PAN RNA, was added to 35 µL (175 µg) of the 0.3 M KCl fraction in a 100 µL total volume. 150 µL bed volume of streptavidin beads was pre-blocked at 4°C for 15 minutes in 100 µg/mL *E. coli* tRNA, 100 µg/mL glycogen and 1 mg/mL BSA, washed three times in 20 mM Tris pH 7.6, 50 mM NaCl and 0.01% NP-40. Extract was nutated with streptavidin beads at 4°C for 1 hour, before pelleting, removing the supernatant, washing beads twice with lysis buffer and eluting bound proteins by treatment with micrococcal nuclease (Worthington Biochemical Corporation) supplemented with 2 mM CaCl_2_ at 37°C for 30 minutes. Proteins were fractionated by 10% SDS PAGE; the PAN RNP-specific bands were excised and identified by mass spectrometry (Columbia University). Further details on all peptides identified in each band can be found at links provided in Fig. S2C.

### Immunofluorescence experiments

BCBL1 TReX-vector or TReX-RTA cells were fixed onto glass slides pre-treated with poly-L-lysine, fixed with 4% paraformaldehyde and permeabilized with 0.5% Triton-X. Anti-PAN RNA oligonucleotides (SB2: ACAAATGCCACCTCACTTTGTCGC; SB85: CGCTGCTTTCCTTTCACATT; SB88: GTGAAGCGGCAGCCAAGGTGACTGG), which were labeled with digoxigenin-dUTP using the DIG oligonucleotide Tailing Kit (Roche), were hybridized with the samples in 50% formamide, 10% dextran sulfate, 2X SSC, 0.1% RNase-free BSA, 500 µg/mL salmon sperm DNA, 125 µg/mL *E. coli* tRNA and 1 mM vanadyl ribonucleoside complexes and detected using FITC-conjugated anti-DIG antibody (Jackson Lab Immunologicals). PABPC1 was visualized with anti-PABPC1 mouse monoclonal or rabbit polyclonal antibodies (gift of G. Dreyfuss, UPenn, and Abcam), and anti-mouse or anti-rabbit Alexa Fluor 594 antibody (Invitrogen). FLAG-PABPC1-NRS, a gift from B. Glaunsinger (UC Berkeley), was visualized with rabbit anti-FLAG antibody (Sigma) and anti-rabbit Alexa Fluor 594 (Invitrogen) [24]. rRNA was visualized with Y10B mouse monoclonal antibody [27] and anti-mouse Alexa Fluor 488 antibody (Invitrogen). Images were collected on a Leica TCS SP5 confocal microscope.

### Modified oligonucleotides

RNase H-targeting oligonucleotide [28], [29] sequences used were as follows. SB215 anti-PAN RNA oligo 1 (complementary to nucleotides 70 to 89 of PAN RNA): mC*mC*mA*mA*mU*G*A*A*A*A*C*C*A*G*A*mA*mG*mC*mG*mG; SB216 anti-PAN RNA oligo 2 (complementary to nucleotides 993 to 1012 of PAN RNA): mU*mG*mA*mG*mC*T*C*T*A*G*G*C*A*C*G*mU*mU*mA*mA*mA; SB230 anti-GFP oligo: mC*mU*mG*mC*mC*A*T*C*C*A*G*A*T*C*G*mU*mU*mA*mU*mC; SB232anti-K7 oligo (complementary to nucleotides -305 to -285 of K7 mRNA, with respect to the PAN RNA transcriptional start site): mA*mA*mU*mC*mG*A*G*C*A*G*A*G*T*A*G*mC*mC*mA*fmA*mG, where m represents 2′-*O*-methyl substitutions of the ribose ring and * represents phosphorothioate substitutions.

### Viral titer assays

BCBL1 TReX-RTA cells were induced with doxycycline for 8 days. Supernatant was collected, passed through a 0.45 micron filter, and incubated with 6.25 µg/mL RNase A (Sigma) and 20 units/mL DNase One (New England Biolabs) for 1 hour at 37°C to degrade extracellular RNA and any viral DNA that had not been properly packaged into viral capsid protein and released into the supernatant. The supernatant was then spun at 15,000 rpm using a Beckman TLA 100.2 rotor for 2 hours at 4°C, and the resulting pellet was resuspended in 600 µL of lysis buffer (20 mM Tris-HCl pH 8.0, 10 mM EDTA, 100 mM NaCl and 0.5% SDS) and incubated at room temperature for 10 minutes to inactivate the DNase. Following proteinase K treatment (0.1 mg/mL final concentration and 2 hours incubation at 37°C), 3 ng of a control plasmid DNA was added to each sample as a normalization control for loss of DNA during subsequent steps of the purification process. After extraction with phenol:chloroform:isoamyl alcohol (25∶24∶1), 600 µL of the aqueous phase was precipitated with isopropanol, sodium acetate and GlycoBlue (Ambion), and viral DNA levels were quantified using qRT-PCR. The viral DNA signal was normalized to signal from the control plasmid DNA (J. Ziegelbauer, personal communication).

For measuring virus production from iSLK.219 cells, cells were induced with doxycycline for 2-3 days, and supernatant was collected and passed through a 0.45 micron filter. 50 µL of supernatant was added to 300 µL of DMEM +10% FBS and placed on 293 DC-SIGN cells that had been grown in 12-well plates. Cells were then spun at 2000 rpm for 1 hour at room temperature in a Beckman Coulter J6-MI centrifuge fitted with a JS-4.2A rotor [30]. 48 hours later cells were harvested by trypsin, washed with PBS and fixed with 4% formaldehyde in PBS for 30 minutes at room temperature. GFP levels were quantified by flow cytometry using BD Biosciences' FACSCalibur platform.

### Intracellular viral DNA replication assays

BCBL1 TReX-RTA cells were electroporated and induced as above for 3 days. 5 million live cells were washed with PBS, resuspended in 250 mM Tris pH 8.5, 125 mM EDTA, 1 mg/mL protease K and 1% SDS, and incubated at 60°C for 2 hours. 1/5th volume of 5 M potassium acetate pH 5.2 was added, lysate was incubated on ice for 20 minutes, and clarified by centrifugation at 12,000 rpm for 15 minutes at 4°C. Supernatant was transferred to a fresh tube, and incubated with 20 µg/mL RNase A at 37°C for 15 minutes. Then, 2.5-fold volume of ice-cold 100% ethanol was added, and DNA was precipitated overnight at −20°C. Pellets were resuspended in 100 µL water, and viral DNA levels were measured by qRT-PCR as above, normalizing to human DNA using GAPDH-specific primers.

## Results

### PABPC1 associates with PAN RNA in the nucleus of BCBL1 TReX-RTA cells

**Figure 1:**
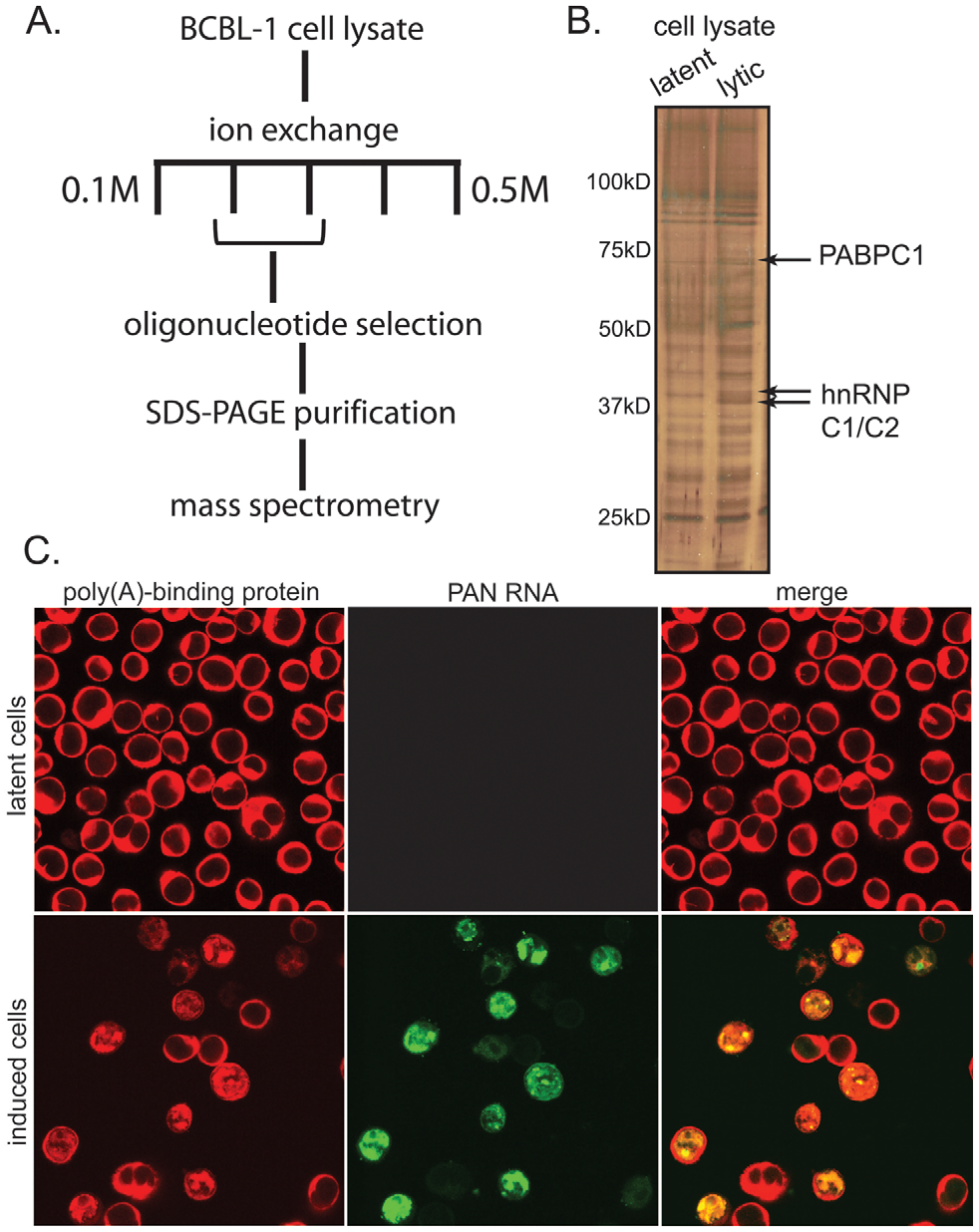
PAN RNA binds to PABPC1 in the nucleus of lytically infected BCBL1 TReX-RTA cells. A. Scheme for biochemical purification of the PAN RNP. B. 10% SDS-PAGE gel silver-stained to show proteins co-purifying with PAN RNA from lysates of doxycycline-treated BCBL1 TReX-vector cells (left lane) or BCBL1 TReX-RTA cells (right lane). C. Dual immunofluorescence and in situ hybridization for PABPC1 (red) and PAN RNA (green) from BCBL1 TReX-vector cells (top panels) or BCBL1 TReX-RTA cells (bottom panels) treated with doxycycline.

To determine what proteins bind to PAN RNA in the nucleus of lytically infected cells, we isolated the PAN RNP from a KSHV-infected cell line containing a doxycycline-inducible version of RTA (BCBL1 TReX-RTA), the viral transcription factor that is necessary and sufficient to promote entry into the lytic phase of viral infection [31], [32], [33]. After anion exchange and affinity chromatography using a selection oligonucleotide complementary to PAN RNA (Fig. 1), we identified both hnRNP C1/C2 and PABPC1 as specifically co-purifying with PAN RNA (see Fig. S1 and S2 for fractionation and mass spectrometry data). These proteins were not seen when extracts from the control doxycycline-treated BCBL1 TReX-vector cell line were used. hnRNP C1 has been previously observed to bind to PAN RNA, although the significance of this interaction is not understood [34].

Since PAN RNA is exclusively nuclear, proteins that interact with PAN RNA must reside in the nucleus. While PABPC1 is essentially a cytoplasmic protein, nuclear re-localization of PABPC1 during KSHV lytic phase had been reported in the literature [18], [19], [20]. We therefore performed immunofluorescence experiments to confirm that PABPC1 re-localizes into the nucleus of the BCBL1 TReX-RTA cells [20] and to establish whether this re-localization correlates with the expression of PAN RNA. Cells were fixed and stained with primary and secondary antibodies to visualize PABPC1, and in situ hybridization probes and detection antibodies to visualize PAN RNA. As seen in Fig. 1C, PABPC1 indeed re-localizes into the nucleus during lytic infection, and the subnuclear distribution of PAN RNA and of re-localized PABPC1 appear strikingly similar. rRNA, however, remains predominantly cytoplasmic during both latent and lytic stages of infection (Fig. S3). We manually scored the immunofluorescence pattern of several hundred cells from a number of microscopic fields, and found that ∼80% of the cells in which PABPC1 had re-localized into the nucleus also expressed PAN RNA (representative images from the manual scoring experiment are provided in Fig. S4). The coincidence of PABPC1 re-localization and PAN RNA expression was further verified in the original BCBL1 cell line (data not shown). High throughput analysis, using multispectral imaging flow cytometry (Amnis Corporation), of PABPC1 nuclear re-localization and PAN RNA expression in BCBL1 cells has extended this correlation (S. Borah, L. Hassman, D.H. Kedes and J.A. Steitz, manuscript in preparation).

Additionally, we noted that unique peptides in the mass spectrometry data also identified PABPC4 as a protein that co-purifies with PAN RNA (Fig. S2A). Recently, nuclear re-localization of this protein has also been reported to result from KSHV lytic infection; knockdown of both PABPC1 and PABPC4 in transient SOX-transfection experiments revealed that these proteins might have redundant functions with respect to SOX-mediated nuclear retention of polyadenylated RNA in 293T cells [24].

### High levels of PAN RNA expression are dependent on the re-localization of PABPC1 into the nucleus, as directed by the SOX protein

**Figure 2:**
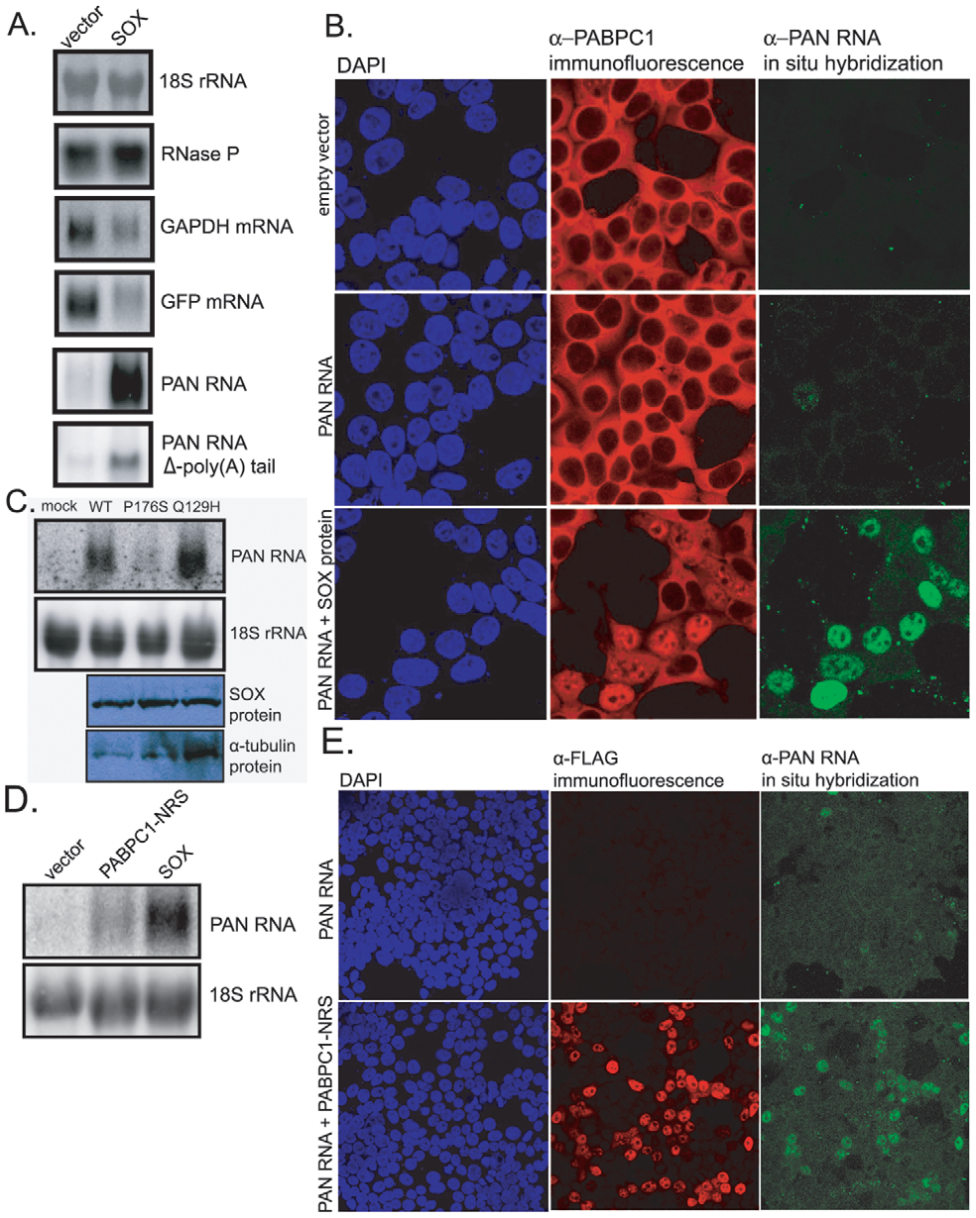
SOX stimulates PAN RNA expression in transient transfection assays. A. Northern blot analysis of total RNA extracted from 293 tet-on cells transfected with: 1) a GFP expression vector, 2) a PAN RNA or PAN/U7 3′-end (PAN RNA Δ-poly(A) tail) expression vector driven from a tetracycline-regulated promoter, and 3) either blank DNA (left lanes) or a SOX-expression vector (right lanes). The blot was sequentially probed for the RNAs listed on the right. B. Confocal images of dual immunofluorescence/in situ hybridization staining for PABPC1 (red) and PAN RNA (green) in transiently transfected 293T cells. Cells were transfected with: 1) empty plasmid (top panels), 2) a PAN RNA expression vector driven from the endogenous RTA-responsive PAN promoter and an RTA expression vector (middle panels), or 3) a PAN RNA expression vector driven from the endogenous RTA-responsive PAN promoter, an RTA expression vector and a SOX-expression vector (bottom panels). C. Top two panels: northern blot analysis for 18S or PAN RNA in total RNA extracted from 293T cells transfected with: 1) PAN RNA driven from its endogenous promoter, 2) an RTA expression vector, and 3) either empty plasmid, an expression vector for wild-type SOX, the P176S mutant SOX or the Q129H mutant SOX. Bottom two panels: western blots showing equal levels of expression of all three SOX proteins. D. Northern blot analysis of total RNA extracted from 293T cells transfected with an RTA expression vector, a PAN RNA expression vector driven from the endogenous PAN promoter and either blank DNA (left lane), a PABPC1-NRS expression vector (center lane) or a SOX expression vector (right lane). E. Confocal images of dual immunofluorescence/in situ hybridization staining for FLAG-tagged PABPC1-NRS (red) and PAN RNA (green) in transiently transfected 293T cells.

Since SOX induces both host shutoff and the nuclear accumulation of PABPC1 [19], [22], and since PAN RNA binds re-localized PABPC1 in the nucleus (see Fig. 1), we hypothesized that PAN RNA might be involved in the host shutoff effect mediated by SOX. To elucidate the relationship between PAN RNA, the SOX protein and the host shutoff effect, we co-expressed PAN RNA and SOX protein in transient transfection experiments, in the absence of other viral genes. As seen in Fig. 2A, transfection of SOX protein into 293 tet-on cells had no effect on 18S rRNA or the RNase P RNA, which are normally unaffected during host shutoff, but resulted in a modest 2- to 4-fold reduction in the level of endogenous GAPDH mRNA and a 2- to 3-fold reduction in the level of co-transfected GFP mRNA. In contrast, transfection of SOX very strongly stimulated the level of PAN RNA (20- to 30-fold). This stimulation was independent of the tetracycline-regulated promoter used to express PAN RNA, as upregulation was observed in experiments using PAN's endogenous RTA-dependent promoter (Fig. 2B and 2C) or a CMV promoter (data not shown).

The effect of SOX on PAN levels was also seen by immunofluorescence (Fig. 2B). When a PAN RNA expression vector that includes 1 kb of PAN's endogenous, RTA-responsive promoter was co-transfected into 293T cells with an RTA expression plasmid, only an occasional in situ hybridization signal for PAN RNA was observed (see Fig. 2B, middle right panel). However, when the SOX gene was co-transfected, the PAN RNA in situ hybridization signal was robust (bottom right panel). Furthermore, those cells in which a robust PAN RNA in situ signal was observed were the same cells in which PABPC1 had re-localized to the nucleus (Fig. 2B, middle column). This suggested that high levels of PAN RNA expression are not only dependent on SOX expression, but might also involve PABPC1 re-localization. Accordingly, when a mutant version of PAN RNA in which the poly(A) tail was replaced by a 3′ stem-loop structure derived from a histone mRNA [6] was transfected into these cells, SOX-mediated enhancement of PAN RNA levels was reduced by about 6-fold (see Fig. 2A, PAN RNA Δ-poly(A) tail, and Fig. S5).

The SOX protein is a dual-function protein, acting both as the mediator of the host shutoff effect and as an alkaline exonuclease that is important for processing the viral DNA [35]. These two functions are separable; point mutants that are unable to carry out the shutoff function are wild-type for exonuclease function, and vice versa. Moreover, mutations that inactivate only the shutoff function of the protein also ablate its ability to re-localize PABPC1 into the nucleus, arguing for a link between these phenomena. We therefore tested each of these mutants to determine which function of SOX is responsible for the strong stimulation of PAN RNA levels. As seen in Fig. 2C, the P176S SOX mutant, which retains exonuclease activity but does not mediate host shutoff, failed to enhance PAN RNA expression. Conversely, the Q129H mutant, which retains host shutoff but does not exhibit exonuclease activity, stimulated PAN RNA expression to the same extent as wild-type SOX. These results indicate that SOX's exonuclease activity is not required for its stimulatory effect on PAN RNA. They are also consistent with the possibility that upregulation of PAN is mediated by SOX's host shutoff function and ability to re-localize PABPC1.

To address whether nuclear localized PABPC1 alone can upregulate levels of PAN RNA, in the absence of SOX, we co-transfected genes for PAN RNA and a mutant PABPC1-NRS that is retained in the nucleus [24]. As seen in Fig. 2D, co-expression of PABPC1-NRS resulted in upregulation of PAN RNA. As with SOX expression, upregulation of PAN RNA levels were specific to those cells in which nuclear retention of PABPC1 had occurred (Fig. 2E). While this result clearly demonstrates that nuclear accumulation of PABPC1 results in increased PAN RNA levels, the levels of PAN RNA did not reach levels seen upon co-transfection with SOX (see Fig. 2D). This may be due to a different efficiency of PABPC1 nuclear re-localization in SOX-transfected versus PABPC1-NRS-transfected cells, or due to some other aspect of SOX expression that further augments PAN RNA expression, in addition to the nuclear localization of PABPC1.

A previous study showed that although SOX and its murine herpesvirus-68 homolog reside in both the nucleus and cytoplasm, it is the cytoplasmic fraction of these proteins that is responsible for the host shutoff effect and for PABPC1 re-localization [36]. A more recent study demonstrated that SOX promotes PABPC1 re-localization by depleting cytoplasmic polyadenylated RNA, which in turn allowed direct intereaction between PABPC1 and importin α [37]. The fact that upregulation of PAN by SOX (Fig. 2A and B) depends on the nuclear re-localization of PABPC1, as well as on the presence of PAN's poly(A) tail, suggests how cytoplasmic SOX protein strongly upregulates the levels of a nuclear RNA: SOX indirectly enhances PAN abundance through re-localizing PABPC1, which in turn stabilizes PAN RNA by binding to its poly(A) tail. This scenario is consistent with the role of poly(A) tails as stabilizing constituents of mRNAs by antagonizing deadenylases [14] and with the presence of nuclear degradation factors that destabilize PAN in the absence of its poly(A) tail [6].

### PAN RNA expression is coincident with but nonessential for the KSHV host shutoff effect

**Figure 3:**
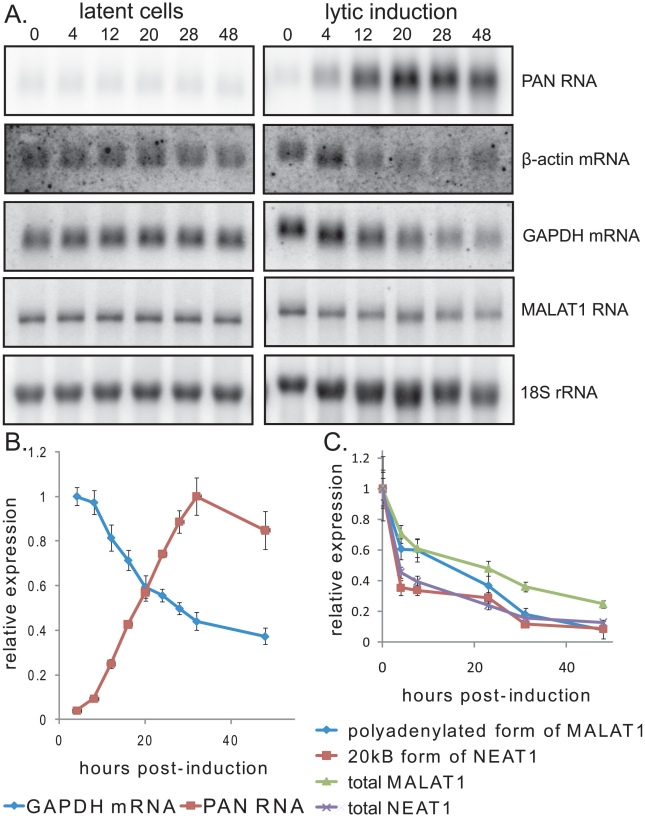
Expression of PAN RNA is coincident with the host shutoff effect. A. Northern blot showing host and viral RNA expression during progression of lytic reactivation by doxycycline treatment of BCBL1 TReX-vector (left) or BCBL1 TReX-RTA (right) cells. B. qRT-PCR data from a representative experiment showing increased expression of PAN RNA versus the decline in host GAPDH mRNA. C. qRT-PCR data from a representative experiment showing that host MALAT1 and NEAT1 RNAs decline during lytic infection. qRT-PCR signals were normalized to endogenous 18S rRNA.

If increased levels of PAN RNA in the nuclei of lytically reactivated cells are due to PABPC1 re-localization, as observed in transiently transfected cells (Fig. 2), then PAN RNA should accumulate at the same time during the course of KSHV infection that host mRNA levels decrease because of the shutoff effect. To test this, BCBL1 TReX-vector or TReX-RTA cells were induced into lytic phase, and total RNA was extracted from culture samples after different times. Northern blot and qRT-PCR analysis (Fig. 3A and B) showed that increases in SOX and PAN RNAs coincide with the decrease in β-actin and GAPDH mRNA (∼12–24 hours post-induction). Thus, PAN RNA expression correlates with the host shutoff effect in the context of actual viral infection. Furthermore, at approximately the same time during the course of lytic infection, PABPC1 re-localizes into the nucleus (data not shown and [19]).

To ask whether lytic induction results in the accumulation of other abundant, polyadenylated noncoding RNAs coincident with PABPC1 re-localization, we measured the levels of host MALAT1 and NEAT1 RNAs by northern blot and qRT-PCR. MALAT1 RNA exists either as a long polyadenylated transcript that localizes to nuclear speckles, or a shorter transcript that localizes to the cytoplasm [38]. Similarly, NEAT1 RNA exists either as a shorter polyadenylated transcript or a longer transcript whose 3′ end is generated by endonucleolytic cleavage by RNase P [39]. We observed that the levels of the nuclear, polyadenylated forms of MALAT1 or NEAT1 RNA did not increase dramatically (Fig. 3A and C) but rather declined in abundance during lytic infection, after normalization to 18S rRNA levels. Thus, PAN RNA selectively accumulates in the nucleus during the KSHV lytic phase, while two other similar noncoding polyadenylated RNAs, MALAT1 RNA and NEAT1 RNA, do not.

**Figure 4:**
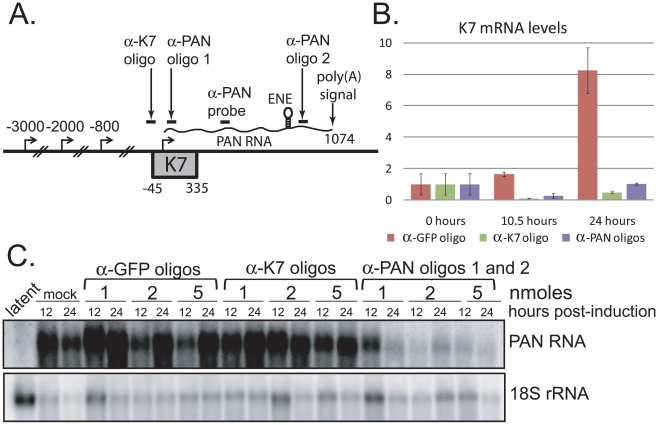
PAN RNA and K7 mRNA levels are reduced by transfection of RNase H-targeting oligonucleotides. A. Region of the KSHV genome from which K7 mRNA and PAN RNA are transcribed. Alternative transcriptional start sites for the K7 mRNA isoforms [40], regions targeted by anti-K7 (α-K7) and anti-PAN RNA (α-PAN) 1 and 2 modified oligonucleotides, as well as the oligonucleotide probe used to detect PAN RNA by northern blot, are indicated. The K7 ORF, ENE and poly(A) signal shared by all four RNA species are shown. B. K7 mRNA levels at 0, 10.5 or 24 hours post-induction of BCBL1 TReX-RTA cells after recovery from electroporation with α-GFP, α-K7 or α-PAN RNA 1 and 2 oligonucleotides. qRT-PCR signals were normalized to endogenous 18S rRNA. C. Northern blot analysis of total RNA extracted from BCBL1 TReX-RTA cells electroporated with different concentrations of α-GFP, α-K7 or α-PAN RNA 1 and 2 oligonucleotides and at 0, 12 or 24 hours post-induction.

To investigate whether PAN RNA expression directly contributes to the host shutoff effect and/or PABPC1 re-localization during the KSHV lytic phase, we knocked down the levels of PAN RNA using 2′-*O*-methylated and phosphorothioate-substituted antisense oligonucleotides that target endogenous RNase H to cleave PAN RNA (α-PAN oligos 1 and 2 in Fig. 4A) [28]. Interpretation of PAN knockdown experiments is complicated by the fact that the region of the KSHV genome from which PAN RNA is transcribed is included in the overlapping K7/survivin transcript (Fig. 4A) [40]. To differentiate between the involvement of PAN RNA and the K7/survivin protein, we designed an additional antisense oligonucleotide that targets the K7 transcript in a region not included in the sequence of PAN RNA (α-K7 oligo in Fig. 4A). We reasoned that any results of transfection of oligonucleotides targeting both PAN RNA and K7 mRNA (Fig. 4A, α-PAN oligos 1 and 2) that are not seen upon transfection of the K7-specific oligonucleotide (α-K7 oligo) would be specifically due to PAN RNA knockdown. First, we showed by qRT-PCR that the K7-specific oligonucleotide selectively lowered the level of the K7 mRNA ∼20-fold (Fig. 4B). However, the K7-specific oligonucleotide did not downregulate levels of PAN RNA, as seen in the northern blot of Fig. 4C. The combination of the two anti-PAN RNA oligonucleotides decreased not only PAN RNA to 5%–10% of wild-type levels in BCBL1 TReX-RTA cells (see Fig. 4C), but also the K7 mRNA, as expected (see Fig. 4B). Thus, differential knockdown of the overlapping K7 and PAN transcripts can be achieved by use of modified oligonucleotides.

**Figure 5:**
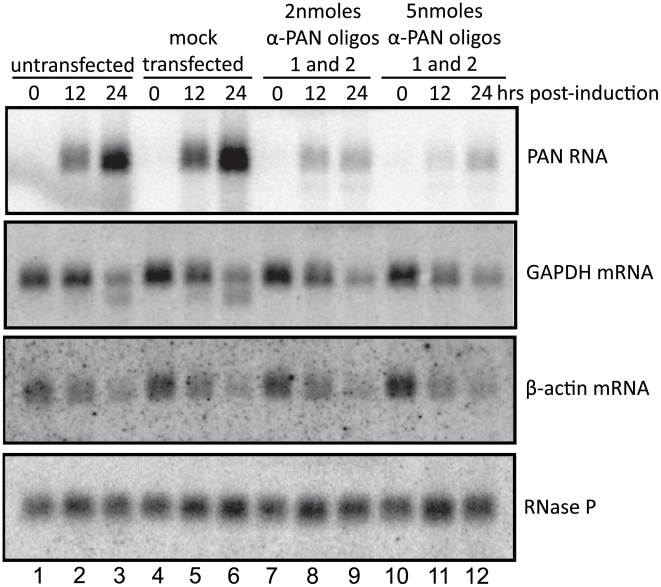
PAN RNA does not contribute to the host shutoff effect. Northern blot of total RNA harvested from BCBL1 TReX-RTA cells electroporated with increasing concentrations of α-PAN RNA 1 and 2 oligonucleotides at various times after doxycycline-mediated induction, probed for the RNAs indicated on the right. The faster migrating band in lanes 3 and 6 of the GAPDH panel is residual ^32^P-labeled anti-PAN RNA probe.

Revealingly, the anti-PAN oligonucleotides had no effect on the host shutoff effect, as indicated by GAPDH and β-actin mRNA levels, despite a knockdown of PAN RNA levels by ∼90%–95% (Fig. 5). Transfection with a GFP- or K7-specific control oligonucleotide also did not alter the magnitude of host shutoff in BCBL1 TReX-RTA cells (Fig. S6). Confocal analysis indicated that PABPC1 was re-localized into the nucleus of BCBL1 TReX-RTA cells upon lytic induction despite PAN RNA knockdown (data not shown). We conclude that the expression of PAN RNA may be related to but is not required for SOX-dependent host shutoff during the KSHV lytic phase.

### PAN RNA is required for expression of a subset of viral genes

Since knockdown of PAN RNA does not appear to affect the SOX-induced shutoff of host protein synthesis, we asked whether PAN RNA might alter the expression of viral proteins instead. We compared the effects of knockdown of PAN RNA on viral protein expression in two different cells lines: BCBL1 TReX-RTA cells, which harbor the authentic KSHV virus, and iSLK.219 cells, in which the recombinant rKSHV.219 virus has been re-introduced into a KS cell line that has lost the original KSHV genome [41], [42]. In both these cell lines, the virus is induced into lytic phase by overexpression of RTA controlled by a tetracycline-responsive promoter.

**Figure 6:**
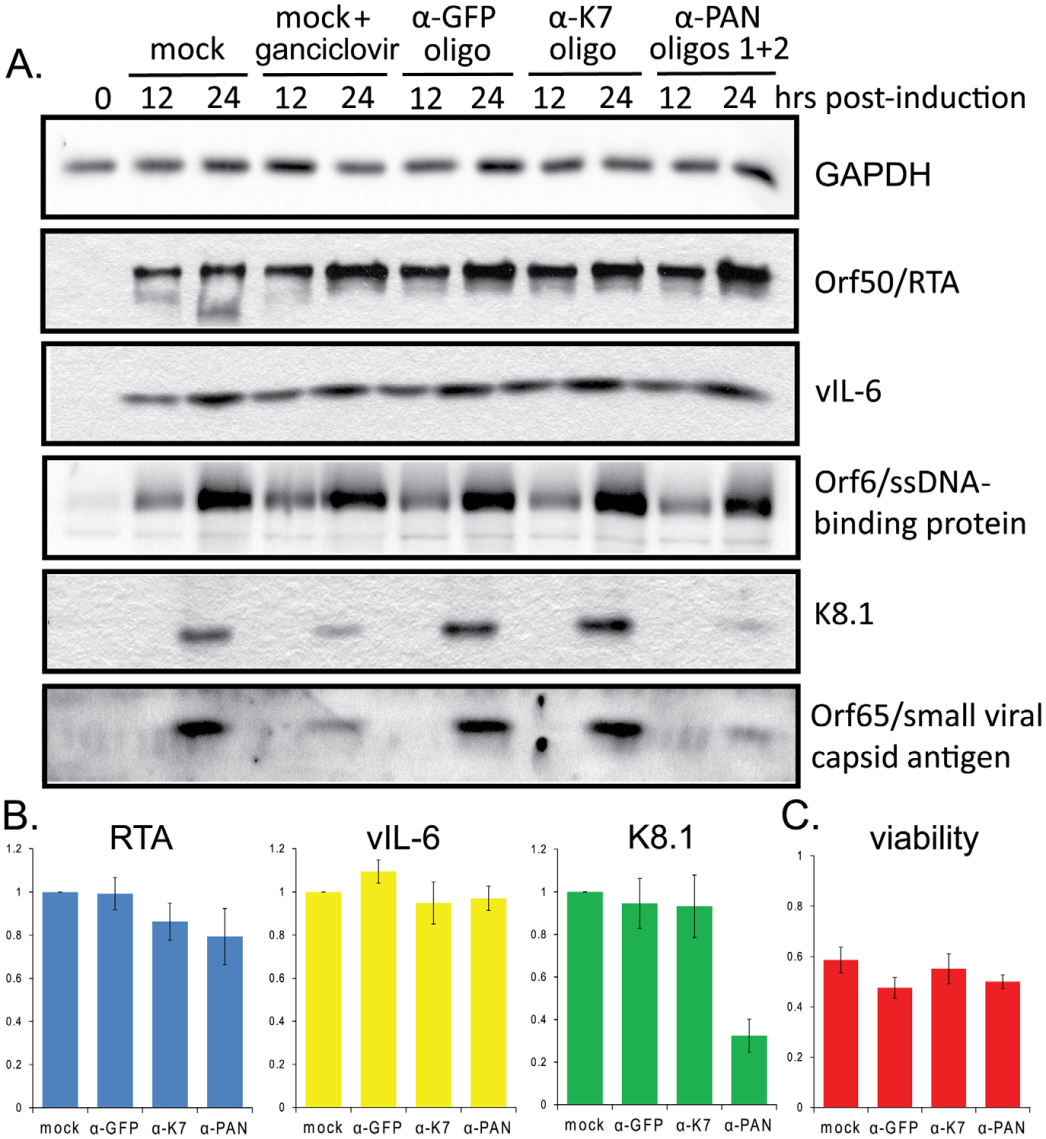
Knockdown of PAN RNA expression adversely affects late gene expression in BCBL1 TReX-RTA cells. A. BCBL1 TReX-RTA cells were electroporated with the indicated modified oligonucleotides and induced with doxycycline 12–16 hours later. After various times, lysates from equivalent numbers of living cells were loaded in each lane, and blotted proteins were probed sequentially with antibodies against the indicated proteins. B. Densitometric analysis of immunoblot signals (see Fig. S6 legend) for RTA (immediate early protein), vIL-6 (early protein) and K8.1 (late protein) after knockdown with control or α-PAN RNA oligonucleotides. Standard error of the mean from 9 experiments (7 experiments for α-K7 knockdown) is shown. C. Cell viability was measured by trypan blue staining 24 hours post-induction. Averages and standard error of the mean from 7 independent experiments are shown.

Western blot analyses of lysates from BCBL1 TReX-RTA that had been transfected with no (mock), anti-GFP, anti-K7 or anti-PAN RNA oligonucleotides revealed that knockdown of PAN RNA affects late rather than early KSHV proteins (Fig. 6). Several early viral proteins, such as Orf50/RTA, vIL-6 and Orf6/ssDNA-binding protein (Fig. 6A), as well as the latency-associated nuclear antigen Orf73/LANA1 (data not shown), accumulate normally. In contrast, the levels of the late viral proteins K8.1 and Orf65/small viral capsid antigen (Fig. 6A), as well as Orf4/complement-binding protein (data not shown) were significantly lowered upon knockdown of PAN RNA, compared to the control oligonucleotides against GFP or K7 mRNAs. Densitometric quantifications by two different methods (see Fig. S7 legend for description of methods) of the immunoblot signals for the immediate early protein RTA, early protein vIL-6 and late protein K8.1 from 9 independent experiments are averaged in Fig. 6B (7 independent experiments for signal from cells transfected with anti-K7 oligonucleotide). The efficiency of PAN RNA knockdown ranged from 75% to 95% (data not shown) and the immunoblots from all 9 experiments are presented in Fig. S7. Together these data demonstrate the selective downregulation of the late protein K8.1 upon knockdown of PAN RNA.

Although non-specific toxic effects were experienced upon electroporation followed by lytic induction, particularly in the BCBL1 TReX-RTA cells (see Materials and Methods), similar levels of cell death were seen regardless of the oligonucleotide transfected (Fig. 6C). Cultures were routinely stained by trypan blue and scored for percent viability prior to harvest in order to analyze lysate from the same number of living cells for each condition. Thus, the loss of late gene expression in PAN knockdown cells is not due simply to increased levels of cell death.

Likewise, several lines of evidence indicate that the decreased levels of late protein expression observed upon knockdown of PAN RNA are not simply due to a non-specific decrease in RTA expression. Although some of the 9 immunoblot experiments revealed a decrease in RTA expression upon transfection with the K7 and PAN RNA oligonucleotides (Fig. 6B), in others a significant decrease in K8.1 expression was observed despite robust expression of RTA (see Fig. S7, panels A, D, E, G and H). Moreover, an independent assay was devised to test whether the effect of PAN RNA knockdown on K8.1 was due to decreased RTA expression. The expression levels of both K8.1 and myc-tagged RTA were measured by dual immunofluorescence staining and analysis by confocal microscopy. Manual scoring of RTA-positive BCBL1 TReX-RTA cells revealed that fewer cells expressed K8.1 upon knockdown of PAN RNA (Fig. S8). Importantly, since K8.1 expression was scored only in cells that also expressed RTA, the observed effect could not be due to decreased RTA expression. We conclude that the effect of PAN RNA knockdown on K8.1 expression is independent of any effect on RTA.

**Figure 7:**
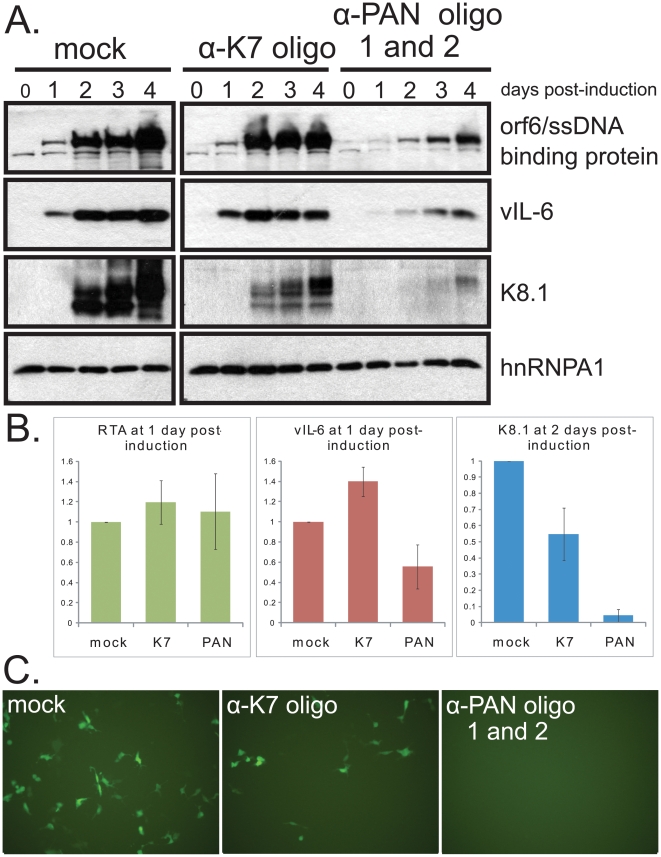
Knockdown of PAN RNA adversely affects gene expression in iSLK.219 cells, with a more pronounced effect on late genes. A. Procedures were the same as in Fig. 6 B. Densitometric analyses of immunoblot signals of RTA (immediate early protein), vIL-6 (early protein) and K8.1 (late protein) after knockdown with control or α-PAN RNA oligonucleotides. Standard error of the mean from 4 experiments is shown. C. GFP expression in target 293 DC-SIGN cells after inoculation with media from iSLK.219 cells treated with no oligonucleotide (mock), α-K7 oligonucleotide or α-PAN RNA oligonucleotides 48 hours after induction with doxycycline. FACS sorting of cells indicated mean fluorescence intensities (in arbitrary units) of 0.07+/− 0.04 for uninfected cells, 1.00 for cells infected with supernatant from mock-transfected iSLK.219 cells, 0.14+/− 0.08 for cells infected with supernatant from anti-K7-transfected iSLK.219 cells and 0.05+/− 0.03 for cells infected with supernatant from anti-PAN-transfected iSLK.219 cells. Standard error of the mean for 3 independent experiments is given.

The results of PAN RNA knockdown in iSLK.219 cells were also assessed (Fig. 7A). Knockdown efficiency in this cell line was 90%–95% (data not shown). In iSLK.219 cells, knockdown of PAN RNA resulted in some decrease in all viral proteins tested, including the early proteins Orf6/ssDNA binding protein and vIL-6 (Fig. 7A). However, the effect on the late K8.1 protein was much more pronounced: K8.1 protein levels dropped 12.5-fold in cells transfected with anti-PAN oligonucleotides compared to cells transfected with anti-K7 oligonucleotide (Fig. 7B), whereas vIL-6 protein levels decreased only 2.5-fold. We conclude that PAN RNA preferentially enhances the expression of late viral proteins in KSHV-infected cells.

### Knockdown of PAN RNA decreases viral titers

**Figure 8:**
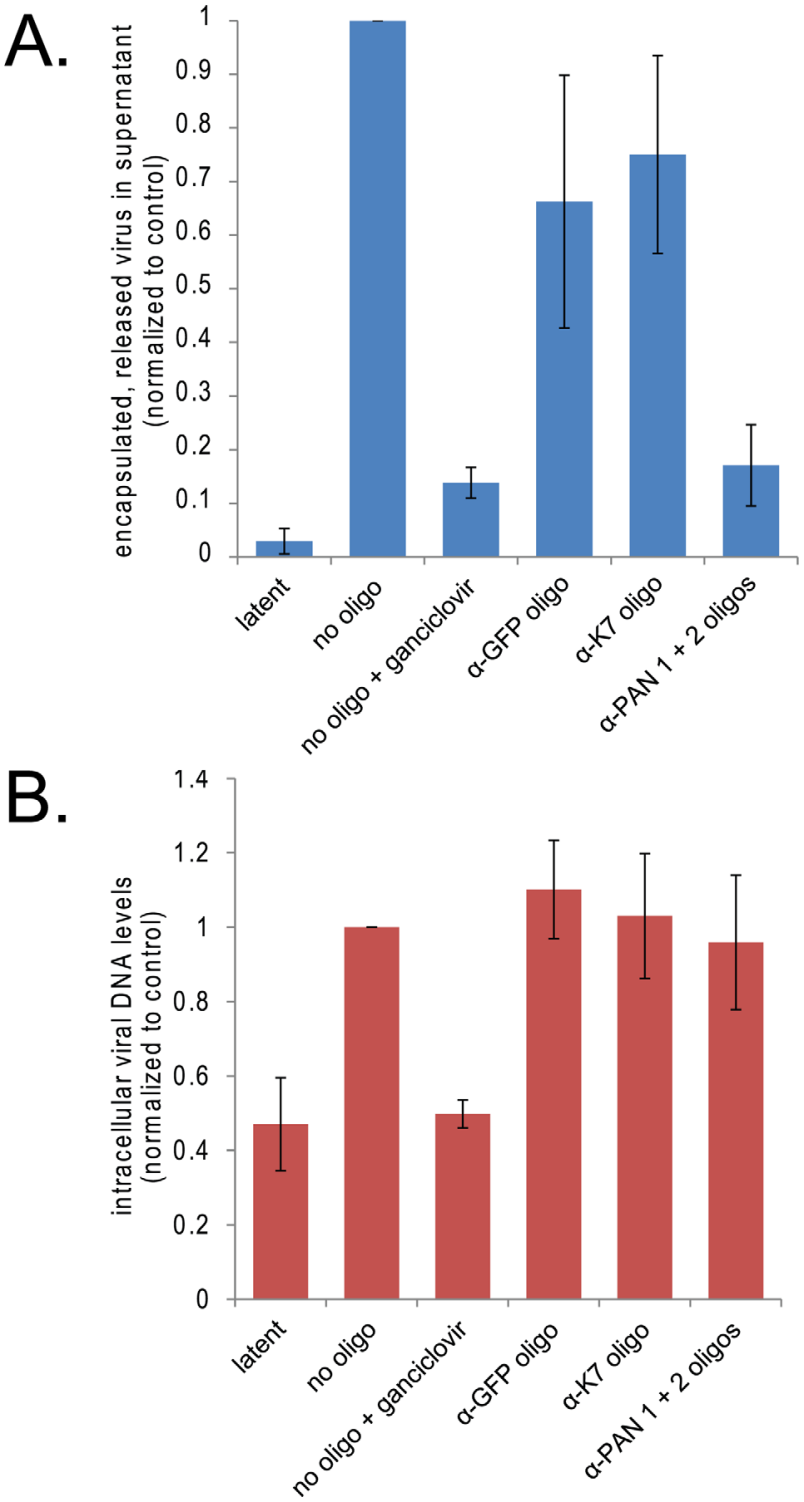
Knockdown of PAN RNA reduces production of encapsulated viral DNA without affecting levels of intracellular viral DNA. A. Knockdown of PAN RNA lowers viral yield in cell culture supernatant. BCBL1 TReX-RTA cells were electroporated with the indicated modified oligonucleotides, allowed to recover overnight, and then induced with doxycycline. 8 days later, encapsulated viral DNA was harvested from the supernatant and quantified by qPCR, normalizing to control plasmid exogenously added at onset of purification. Values are the average of 4 independent experiments. Standard error of the mean is shown. Note that electroporation of all oligonucleotides decreased virus production to some degree, most probably because of cell death experienced upon electroporation of nucleic acids followed by lytic induction. B. Knockdown of PAN RNA does not decrease intracellular viral DNA. BCBL1 TReX-RTA cells were treated as above and intracellular DNA was collected 3 days post-induction and quantified by qPCR, normalizing to the amount of host DNA, as measured by GAPDH DNA signal. Values are the average of 3 independent experiments. Standard error of the mean is shown.

Since knockdown of PAN RNA adversely affects expression of important late viral genes, we predicted that its knockdown would affect overall viral yield. To assess the role of PAN RNA in the production of new virus, we harvested virus from the supernatant of induced BCBL1 TReX-RTA cells 8 days post-induction. As seen in Fig. 8A, knockdown of PAN RNA significantly reduced viral production, as assayed by qPCR measurement of DNase resistant, encapsulated viral DNA released into the supernatant. The effect was comparable to treatment with ganciclovir, an inhibitor of viral DNA replication [43], [44].

The effect of PAN RNA knockdown on production of infectious virus from iSLK.219 cells was also assayed by harvesting the supernatant from induced iSLK.219 cells and infecting target 293 cells that stably express the DC-SIGN receptor. Infection was assessed by visualization (Fig. 7C) and by FACS analysis of GFP expression arising from the recombinant KSHV genome in target cells 48 hours later. Fig. 7C reveals that treatment of iSLK.219 cells with anti-PAN RNA oligonucleotides resulted in virtually no GFP-positive 293 DC-SIGN cells upon infection with virus harvested from cells treated with anti-PAN oligonucleotides. However, GFP-positive cells were detected among 293 DC-SIGN cells incubated with virus harvested from cells treated with the anti-K7 oligonucleotide. Thus, release of infectious virus as well as encapsulated viral DNA levels were both diminished by knocking down PAN RNA.

Since viral DNA replication is required for the expression of herpesvirus late proteins [45], [46], [47], the effect of PAN RNA knockdown on the accumulation of intracellular viral DNA during the lytic phase was also measured by qPCR. Increases in intracellular viral DNA levels 4 days post-induction in BCBL1 TReX-RTA cells were very modest (∼5–7 fold), in agreement with other reports [48], [49]; this level was further decreased when cells were electroporated prior to induction. The low levels of intracellular viral DNA make it difficult to conclude definitively whether the knockdown of PAN RNA specifically inhibits viral DNA replication. However, no dramatic differences in intracellular viral DNA levels were detectable in cells transfected with control versus anti-PAN RNA oligonucleotides (Fig. 8B), suggesting that PAN RNA is not directly involved in viral DNA replication.

## Discussion

### A noncoding RNA is important for viral gene expression during the KSHV lytic phase

The data presented here indicate that the highly abundant PAN RNA binds to the normally cytoplasmic poly(A) binding protein PABPC1 once it has been re-localized to the nucleus of KSHV-infected cells (Fig. 1). This is supported by both the composition of the PAN RNP and the observation that the detailed patterns of PAN RNA and PABPC1 concentration within the nucleus coincide. The abundance of PAN RNA (upwards of 0.5×10^6^ transcripts per nucleus) corresponds well to the abundance of PABPC1 (estimated at 7×10^6^ copies in the average HeLa cell), given that the length of PAN RNA's poly(A) tail is estimated to be similar to that of the average host mRNA (Conrad and Steitz, data not shown) and that each PAN RNA thus likely binds 8–10 PABPC1 molecules [3], [50]. Our results extend prior reports of PABPC1 re-localization by SOX protein alone and the concurrent host shutoff effect [19] by showing that that the level of PAN RNA is likewise dependent on expression of the SOX protein in transient transfection assays (Fig. 2). In infected cells, PAN RNA is not required for the re-localization of PABPC1 nor for the host shutoff effect (Fig. 5), which promotes this re-localization.

Since PAN RNA expression is concurrent with the host shutoff effect in the context of viral lytic infection (Fig. 3), PAN RNA likely functions downstream of SOX action. An alternative possibility-that RTA levels (and therefore all late functions) are compromised by treatment with anti-PAN oligonucleotides-is not consistent with several observations. Because lytic reactivation is dependent on RTA expression, an across-the-board reduction in all lytic-related events would be expected but was not observed. Specifically, PAN RNA knockdown did not affect: 1) the host shutoff effect and PABPC1 nuclear re-localization (Figs. 5 and S6 and data not shown), 2) accumulation of intracellular viral DNA (Fig. 8B), and 3) expression of the viral lytic marker vIL-6 (Fig. 6A, B). Instead, knockdown of PAN RNA adversely affected the expression of late viral genes in cells of both lymphoid (BCBL1, Figs. 6, S7 and S8) and endothelial (iSLK.219, Fig. 7) origin, which are two major KSHV targets in vivo.

Ultimately, knockdown of PAN RNA adversely affects release of new infectious virus into the supernatant from BCBL1 TReX-RTA cells (Fig. 8A), as would be expected from the downregulation of late viral gene expression. This effect appears to be independent of viral DNA replication (Fig. 8B). We conclude that PAN RNA plays an important role in the expression of a subset of viral genes, perhaps related to the nuclear re-localization of PABPC1. Further work is needed to establish whether this effect is at the level of transcription, translation or mRNA stability, although selective downregulation of mRNAs for the late genes K8.1, Orf18 and Orf29 was observed using qRT-PCR analysis (data not shown). It is also possible that the effects seen on virus production and late viral protein expression result from dual knockdown of both PAN RNA and K7. However, the fact that the knockdown of PAN RNA is required to observe these effects, as K7 knockdown alone is insufficient, underscores the importance of PAN RNA.

KSHV targets host gene expression at several levels during the lytic phase. First, host mRNA levels are specifically downregulated by the SOX protein, a phenomenon that is itself related to nuclear re-localization of PABPC1, a highly abundant translation factor that displays nanomolar affinity for the polyadenylate tails of mRNAs [24], [50]. Rowe and colleagues have found that the Epstein Barr virus SOX homolog BGLF5 mediates host shutoff in EBV-infected cells [51], and Glaunsinger and colleagues have extended their findings of SOX function to the homologous murine herpesvirus-68 (MHV-68) protein [36]. Both EBV and MHV-68 SOX homologs drive PABPC1 re-localization into the nucleus, like their KSHV-counterpart [19], and we have extended the observation of PABPC1 nuclear re-localization to EBV-infected HH514-16 cells upon lytic activation (S. Borah, R. Park, G. Miller and J.A. Steitz, unpublished observations). Second, in addition to the downregulation of host mRNA levels, other changes in host translation during the KSHV lytic phase [20] have been reported to include increased levels of 4E-BP1 and eIF4E phosphorylation, which are expected to enhance rates of eIF4F assembly onto and of translation of viral mRNAs. Third, KSHV mRNAs, many of which are unspliced, are preferentially exported by the viral export factor Orf57, which tethers the nuclear export factor TAP protein to viral mRNAs and enhances their translation [52], [53], [54].

Our data support a model in which the highly abundant PAN RNA contributes importantly to the viral manipulation of gene expression. Aspects of viral infection that downregulate host gene expression, such as host shutoff and PABPC1 re-localization, do not require PAN RNA, but instead affect the expression level of PAN RNA itself both in transiently transfected cells and in bona fide infected cells. PAN RNA then impacts the expression of at least a subset of viral genes; possible molecular mechanisms for this regulation are presented below.

### Viral targeting of PABP

Poly(A) binding protein is a central player in cellular gene expression and is targeted by a number of viruses, usually as a means to achieve shutdown of host gene expression. Some, such as picornaviruses and caliciviruses, have evolved specific proteases that cleave PABPC1 [55], [56], [57]. Rotavirus instead expresses the NSP3 protein, which displaces PABPC1 from binding to eIF4G. This reduces the translational efficiency of host mRNAs, whereas viral mRNAs, which lack a poly(A) tail, are efficiently translated [58]. Expression of NSP3 also leads to nuclear accumulation of PABPC1, although the significance of this re-localization is not fully understood [59], [60]. Nuclear re-localization of PABPC1 is also observed during infection with Bunyamwera virus. Furthermore, although siRNA-mediated knockdown of PABPC1 decreased translation of a polyadenylated luciferase reporter, translation of a reporter whose 3′ end was derived from Bunyamwera virus mRNA, and thus lacked a poly(A) tail, was unaffected by knockdown [61].

PABPN1, a distinct nuclear poly(A) binding protein [14], is also targeted by viruses. Influenza NS1 protein binds both PABPN1 and the cleavage and polyadenylation stimulation factor (CPSF) within the assembled 3′-end processing machinery [62]. Thus, poly(A) polymerase does not processively extend the poly(A) tail of nascent mRNAs past ∼12 residues, and multiple PABPN1 molecules do not assemble onto the mRNA. This failure to properly form a poly(A) tail [63] and interact with PABPN1 are thought to underlie the lack of host mRNA export during influenza virus infection [64]. NS1 has other effects on PABPN1 as well, including inhibition of PABPN1 shuttling and re-distribution of PABPN1 from nuclear speckles to a uniform pattern throughout the nucleoplasm [62]. However, to date, no role for a noncoding RNA has been identified in the mechanisms by which these viruses target PABPC1 for host shutoff.

### Homologs of PAN RNA?

Given the striking abundance of PAN RNA and the role that it plays in KSHV gene expression within the context of PABPC1 re-localization, it might be expected that homologs of this RNA would be found in related herpesviruses in which re-localization of PABPC1 occurs. Viruses whose mRNAs lack poly(A) tails, such as rotavirus and Bunyamwera virus, would be exceptions. Indeed, the EBV homolog of the SOX protein, BGLF5, has been shown to mediate host shutoff in cells infected with EBV and to drive PABPC1 re-localization [36], [51]. Although no PAN-like RNA has yet been reported in EBV or other herpesviruses, we have recently discovered putative homologs in several members of the gamma herpesvirus family. Indeed, expression of a PAN homolog has been verified in the closely related Rhesus Rhadinovirus (RRV), even though the overlapping K7 open reading frame does not appear to be conserved (K. Tycowski, S. Borah, M. Shu and J.A. Steitz, manuscript in preparation) [65], [66]. Additional studies are required to explore the possibility that PAN RNA-like RNAs also exist in more distantly-related viruses such as EBV and MHV-68. Interestingly, the sequence of PAN-like RNAs may not be conserved, whereas their abundance, nuclear localization and the presence of a poly(A) tail may be critical for function. Furthermore, there is no reason to expect that the role of PAN RNA be fulfilled by a single RNA transcript. Perhaps in other viruses, PAN RNA is functionally replaced by two or three different polyadenylated, nuclear RNAs, which together sequester PABPC1. Immunoprecipitation of PABPC1 from the nuclei of other herpesviruses during the lytic phase and sequencing the RNAs that co-precipitate might identify such PAN-like RNAs.

Interestingly, the best-characterized viral noncoding RNAs, the VA RNAs of adenovirus, which facilitate viral translation by suppressing protein kinase R and competitively inhibiting miRNA export and processing [67], [68], are not found outside of adenoviruses. Perhaps their biological functions are fulfilled by alternative mechanisms in other viruses.

### Potential molecular mechanisms for the role of PAN RNA in the nucleus

By what molecular mechanism might PAN RNA enhance the expression of late viral genes, and could this role be related to its ability to bind re-localized PABPC1 in the nucleus of KSHV-infected cells? It is first critical to understand why PABPC1 re-localization might be harmful to the cell's normal functioning. PABPC1 is proposed to have three major functions: 1) to synergistically enhance mRNA translation by interaction with eIF4G, 2) to regulate mRNA fate by inhibiting deadenylation of the poly(A) tail or promoting interaction with deadenylating factors, and 3) to facilitate nuclear export of mRNA [14]. It seems likely that loss of PABPC1 from the cytoplasm would be detrimental to any or all of these functions in uninfected cells. In the context of viral infection, re-localization of PABPC1 appears to be important for the shutoff effect since siRNA-mediated knockdown of PABPC1 diminishes the ability of SOX to target GFP mRNA for degradation, and since point mutations in SOX that ablate its effect on PABPC1 re-localization also abolish its downregulation of host mRNAs [19]. Importantly, aspects of host shutoff have been recapitulated by transient transfection of a nuclear targeted PABPC1 protein [24]. These results argue that it is not merely the absence of PABPC1 from the cytoplasm that is detrimental for host mRNA stability, but that its presence in the nucleus is pivotal.

**Figure 9:**
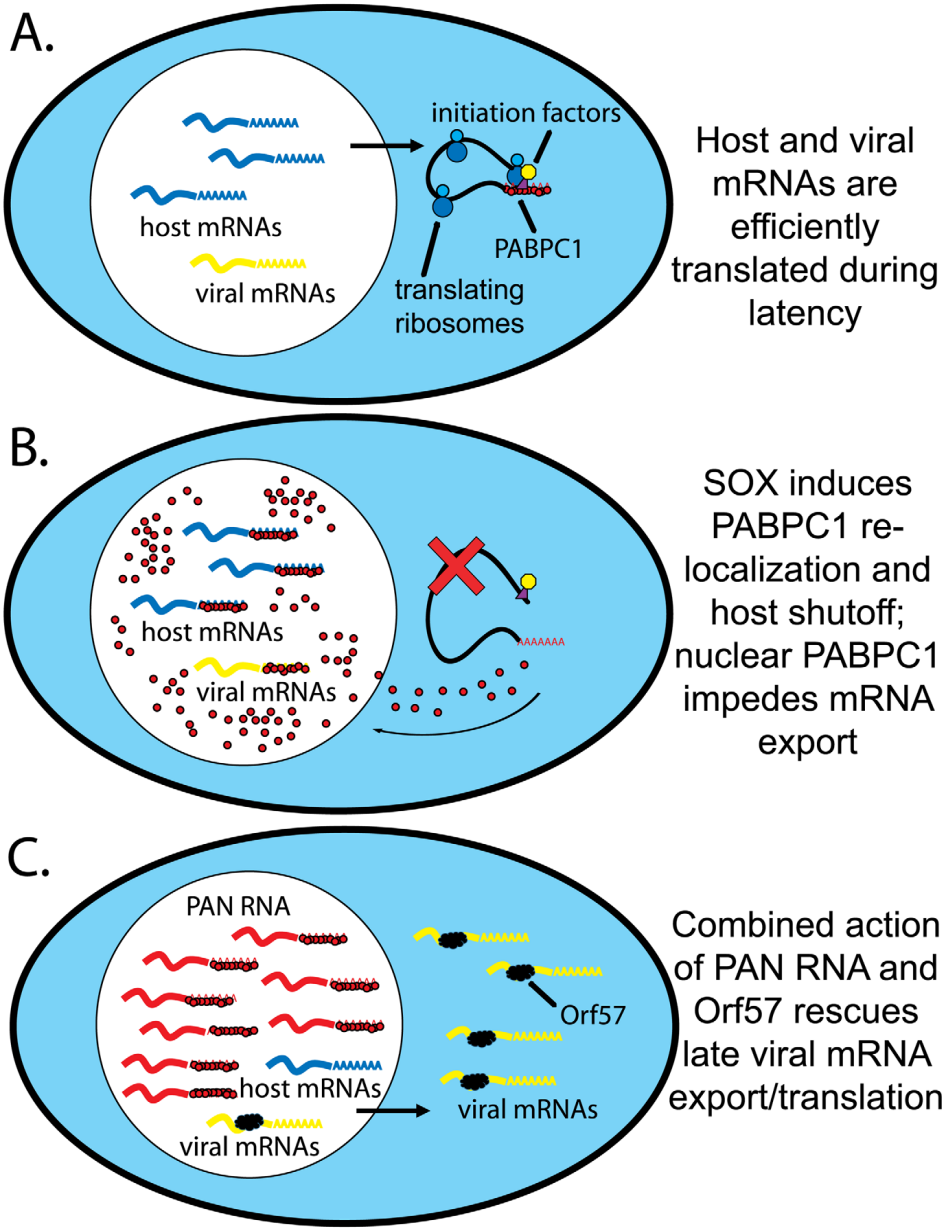
Model for PAN RNA sequestering nuclear re-localized PABPC1. A. mRNA transcription, export and translation during latent infection. Host mRNAs (blue) are exported and bound by initiation factors and PABPC1. B. Viral infection in the absence of PAN RNA. SOX action mediates host shutoff and PABPC1 nuclear re-localization. Host and viral mRNAs are bound by PABPC1, leading to inefficient processing and/or export and resulting degradation. C. Viral infection in the presence of PAN RNA. As in B, PABPC1 is re-localized but bound by PAN RNA. Viral, but not host, mRNAs are bound by Orf57, which mediates their efficient export into the cytoplasm.

How might nuclear re-localized PABPC1 contribute to host mRNA degradation? Under normal conditions, PABPN1 is required for the synthesis of and co-transciptionally binds to the newly-extended poly(A) tails of mRNAs. It has been suggested that PABPN1 is further required for proper export of mRNA via its interaction with nuclear export factors [63]. PABPC1, which shuttles in and out of the nucleus, is also thought to aid in mRNA export by interacting with polyadenylated mRNA at an early stage in maturation [69], [70], [71], [72], [73]. The dramatic influx of PABPC1 into the nucleus during KSHV lytic infection might therefore perturb the process of mRNA nuclear export. Given the extreme abundance of PABPC1 and its higher affinity for poly(A) tails, nuclear PABPC1 (K_d_ ∼7 nM) could displace PABPN1 (K_d_ ∼555 nM) from polyadenylated mRNAs and interfere with PABPN1's role in export [50], [74]. As mRNAs that are not properly processed and exported from the nucleus become hyperadenylated and degraded [19], [75], [76], [77], the re-localization of PABPC1 to the nucleus could be the initiating step in their degradation during shutoff (Fig. 9A and B). Similar ideas are discussed in two recent studies, by Glaunsinger and colleagues, on the inhibition of nuclear PABPC1 on mRNA export [24], [37].

If a highly abundant, inert, polyadenylated RNA were expressed in the nucleus, then the re-localized PABPC1 would be stoichiometrically bound, or nearly so. Thus, PAN RNA could serve as a “buffer” against changes in nuclear PABPC1 concentration (Fig. 9C). The fact that host-cell encoded abundant, nuclear polyadenylated RNAs, such as MALAT1 and NEAT1 RNA, do not accumulate to exceptionally high levels in response to PABPC1 re-localization (Fig. 3) suggests that there is something unique about PAN RNA. It should be noted that PABPN1 knockdown also diminishes SOX-mediated mRNA degradation, so it may be the balance of the two proteins on the poly(A) tail that is critical [19]. A comparison of intranuclear PABPC1 and PABPN1 immunofluorescence signals relative to PAN RNA in situ hybridization signal should be performed.

A challenge for the model (Fig. 9) is explaining how PAN RNA specifically protects viral mRNAs. Should not host mRNAs be equally protected from the negative effects of PABPC1 re-localization by the abundant expression of PAN RNA? Some possible explanations derive from fundamental differences between viral and host mRNAs in the nucleus. First, the viral Orf57 protein selectively binds intronless viral mRNAs, mediating their export by anchoring the human transcription and export complex (hTREX) [52]. Since most KSHV genes lack introns, Orf57 is not only important, but in fact essential for virus replication [53]. Second, transcription rates of viral mRNAs appear to greatly outpace those of host mRNAs during the lytic phase [23]. Thus, viral mRNAs may be preferentially exported and translated during the late lytic phase simply because they outcompete their host counterparts. PAN RNA would therefore function in concert with several other proteins (PABPN1 and PABPC1, SOX and Orf57) to create an environment that favors the export and expression of viral but not host mRNAs (Fig. 9C).

## Supporting Information

Figure S1 Induction of PAN RNA in BCBL1 TReX-RTA cells and purification of the PAN RNP from cell lysate. A. Northern blot of total RNA harvested from BCBL1 TReX-RTA cells treated with doxycycline for the indicated number of hours. B. Northern blot of fractions collected during purification of the PAN RNP by anion exchange and affinity chromatography steps from lysate prepared at 24 hours post-induction.
(EPS)

Figure S2 Proteins identified by mass spectrometry, including PABPC1 (top band), hnRNPC1 and hnRNPC2 (middle and bottom bands), as co-purifying with PAN RNA. A. List of proteins identified and the number of unique peptides identified for each protein in each band. B. Silver-stained gel showing better resolution of the top band, containing PABPC1, from a closely migrating non-specific band, and a zoomed-in image of the silver-stained gel in Fig. 1B showing the hnRNPC1 and C2 bands. C. Links to complete mass spectrometry data for all three protein bands.
(EPS)

Figure S3 Confocal images of PABPC1 (red) and rRNA (green) in latent or lytic BCBL1 TReX-vector and TReX-RTA RTA cells, respectively. Both cell lines were treated with 1 µg/mL doxycycline for 24 hours before fixation and staining with anti-PABPC1 and Y10B anti-rRNA antibodies. Red and green channels merge into yellow in the cytoplasm of latently infected cells, showing that both PABPC1 and rRNA are predominantly cytoplasmic during latency. However, while PABPC1 (red) re-localizes into the nucleus during lytic phase, rRNA (green) remains cytoplasmic.
(EPS)

Figure S4 Manual scoring of images taken with a laser scanning confocal microscope indicates that PAN RNA expression and PABPC1 re-localization are highly correlated. Left panels show wide field images of PABPC1 (top, red) and PAN RNA (bottom, green). Right panels show these same images, except that saturated pixels are identified in white and background pixels are in gray. Saturated and background signals aided scoring since cytoplasmic PABPC1 stained very intensely compared to re-localized PABPC1 and since the anti-PAN RNA oligonucleotide probes described in Materials and Methods sometimes gave significant background. Images of representative regions in the lower 4 panels are indicated by white boxes in the upper 4 panels. A total of 450 cells were scored independently by two individuals. The percent of cells with re-localized PABPC1 that also expressed PAN RNA was scored as 73% by person 1 (larger sample size), and 90% by person 2 (smaller sample size), for an average score was 76%.
(TIF)

Figure S5 Replacing the poly(A) signal of PAN RNA with a U7 snRNP-dependent 3′-end formation signal (PAN RNA Δ-poly(A) tail) [6] reduces the effect of SOX co-transfection on PAN accumulation in 293T cells, as viewed by confocal microscopy. Note that some cells in which PABPC1 appears nuclear show slightly enhanced signal even for PAN RNA lacking a poly(A) tail, as indicated with white arrows, consistent with the northern blot results (see Fig. 2A).
(TIF)

Figure S6 qRT-PCR analysis of GAPDH mRNA in total RNA extracted from BCBL1 TReX-RTA cells that were electroporated with the indicated modified oligonucleotides and induced with doxycycline for the indicated times.
(EPS)

Figure S7 Immunoblots probed with anti-RTA, anti-vIL-6 and anti-K8.1 antibodies, from 9 independent knockdown experiments. Blots were developed either with a G:BOX (Syngene) (panels A, B, C and E) or by x-ray film (panels D, F, G, H and I). For the former, densitometry analysis was performed with GeneTools software (Syngene), ensuring use of non-saturated signal. Furthermore, linearity of signal was confirmed by titration of lysate. For data collected with x-ray film, a titration of exposure time was performed for each blot to avoid analysis of x-ray film that had been overexposed. X-ray films were then scanned and densitometry was performed using Image J software (NIH). Average RTA, vIL-6 and K8.1 signal from cells treated with mock, anti-GFP, anti-K7 and anti-PAN RNA oligonucleotides are provided in Figure 6B.
(EPS)

Figure S8 Manual scoring of RTA and K8.1 expression in BCBL1 TReX-RTA cells electroporated with no oligonucleotide (mock), anti-GFP, anti-K7 or anti-PAN oligonucleotides. A. Representative images from those used to score RTA and K8.1 expression in latently infected (top) and lytically infected (bottom) cells. First, DAPI was used to identify healthy cells correctly positioned on the z-axis (panels marked DAPI). Then, RTA-myc expression was scored by overlaying the Alexa Fluor 594 signal (panels marked DAPI+RTA). Finally, K8.1 expression was scored by overlaying the Alexa Fluor 488 signal (panels marked DAPI+RTA+K8.1). B. Quantification of 4 independent experiments (except for the anti-K7 oligonucleotide transfection, where n = 3). 10 randomly chosen fields, each containing about 60–70 cells, were scored per treatment for each experiment. Standard error of the mean is shown, and Student's t-test values are as follows: anti-GFP versus anti-K7 = 0.987, anti-GFP versus anti-PAN = 0.023, anti-K7 versus anti-PAN = 0.045. For one of these 4 experiments, a second person independently scored K8.1 and RTA expression, confirming a significant decline in K8.1 expression comparing anti-K7 and anti-PAN treated cells (Student's t-test = 0.009). The difference between K8.1 expression in anti-GFP versus anti-PAN treated cells was less significant, as individually scored by this person (Student's t-test = 0.105).
(EPS)

## References

[1] Verma SC, Robertson ES (2003). Molecular biology and pathogenesis of Kaposi sarcoma-associated herpesvirus.. FEMS Microbiol Lett.

[2] Ganem D (2006). KSHV infection and the pathogenesis of Kaposi's sarcoma.. Annu Rev Pathol.

[3] Sun R, Lin SF, Gradoville L, Miller G (1996). Polyadenylylated nuclear RNA encoded by Kaposi sarcoma-associated herpesvirus.. Proc Natl Acad Sci U S A.

[4] Zhong W, Ganem D (1997). Characterization of ribonucleoprotein complexes containing an abundant polyadenylated nuclear RNA encoded by Kaposi's sarcoma-associated herpesvirus (human herpesvirus 8).. J Virol.

[5] Conrad NK, Steitz JA (2005). A Kaposi's sarcoma virus RNA element that increases the nuclear abundance of intronless transcripts.. EMBO J.

[6] Conrad NK, Mili S, Marshall EL, Shu MD, Steitz JA (2006). Identification of a rapid mammalian deadenylation-dependent decay pathway and its inhibition by a viral RNA element.. Mol Cell.

[7] Conrad NK, Shu MD, Uyhazi KE, Steitz JA (2007). Mutational analysis of a viral RNA element that counteracts rapid RNA decay by interaction with the polyadenylate tail.. Proc Natl Acad Sci U S A.

[8] Pawlicki JM, Steitz JA (2008). Primary microRNA transcript retention at sites of transcription leads to enhanced microRNA production.. J Cell Biol.

[9] Mitton-Fry RM, DeGregorio SJ, Wang J, Steitz TA, Steitz JA (2010). Poly(A) tail recognition by a viral RNA element through assembly of a triple helix.. Science.

[10] Bernstein P, Peltz SW, Ross J (1989). The poly(A)-poly(A)-binding protein complex is a major determinant of mRNA stability in vitro.. Mol Cell Biol.

[11] Ford LP, Bagga PS, Wilusz J (1997). The poly(A) tail inhibits the assembly of a 3′-to-5′ exonuclease in an in vitro RNA stability system.. Mol Cell Biol.

[12] Korner CG, Wormington M, Muckenthaler M, Schneider S, Dehlin E (1998). The deadenylating nuclease (DAN) is involved in poly(A) tail removal during the meiotic maturation of Xenopus oocytes.. EMBO J.

[13] Wilusz CJ, Gao M, Jones CL, Wilusz J, Peltz SW (2001). Poly(A)-binding proteins regulate both mRNA deadenylation and decapping in yeast cytoplasmic extracts.. RNA.

[14] Mangus DA, Evans MC, Jacobson A (2003). Poly(A)-binding proteins: multifunctional scaffolds for the post-transcriptional control of gene expression.. Genome Biol.

[15] Tarun SZ, Sachs AB (1996). Association of the yeast poly(A) tail binding protein with translation initiation factor eIF-4G.. EMBO J.

[16] Tarun SZ, Wells SE, Deardorff JA, Sachs AB (1997). Translation initiation factor eIF4G mediates in vitro poly(A) tail-dependent translation.. Proc Natl Acad Sci U S A.

[17] Wells SE, Hillner PE, Vale RD, Sachs AB (1998). Circularization of mRNA by eukaryotic translation initiation factors.. Mol Cell.

[18] Kanno T, Sato Y, Sata T, Katano H (2006). Expression of Kaposi's sarcoma-associated herpesvirus-encoded K10/10.1 protein in tissues and its interaction with poly(A)-binding protein.. Virology.

[19] Lee YJ, Glaunsinger BA (2009). Aberrant herpesvirus-induced polyadenylation correlates with cellular messenger RNA destruction.. PLoS Biol.

[20] Arias C, Walsh D, Harbell J, Wilson AC, Mohr I (2009). Activation of host translational control pathways by a viral developmental switch.. PLoS Pathog.

[21] Glaunsinger B, Ganem D (2004). Highly selective escape from KSHV-mediated host mRNA shutoff and its implications for viral pathogenesis.. J Exp Med.

[22] Glaunsinger B, Ganem D (2004). Lytic KSHV infection inhibits host gene expression by accelerating global mRNA turnover.. Mol Cell.

[23] Chandriani S, Ganem D (2007). Host transcript accumulation during lytic KSHV infection reveals several classes of host responses.. PLoS ONE.

[24] Kumar GR, Glaunsinger BA (2010). Nuclear import of cytoplasmic poly(A) binding protein restricts gene expression via hyperadenylation and nuclear retention of mRNA.. Mol Cell Biol.

[25] Bryan BA, Dyson OF, Akula SM (2006). Identifying cellular genes crucial for the reactivation of Kaposi's sarcoma-associated herpesvirus latency.. J Gen Virol.

[26] McAllister SC, Hansen SG, Messaoudi I, Nikolich-Zugich J, Moses AV (2005). Increased efficiency of phorbol ester-induced lytic reactivation of Kaposi's sarcoma-associated herpesvirus during S phase.. J Virol.

[27] Lerner EA, Lerner MR, Janeway CA, Steitz JA (1981). Monoclonal antibodies to nucleic acid-containing cellular constituents: probes for molecular biology and autoimmune disease.. Proc Natl Acad Sci U S A.

[28] Ideue T, Hino K, Kitao S, Yokoi T, Hirose T (2009). Efficient oligonucleotide-mediated degradation of nuclear noncoding RNAs in mammalian cultured cells.. RNA.

[29] Sasaki YT, Ideue T, Sano M, Mituyama T, Hirose T (2009). MENepsilon/beta noncoding RNAs are essential for structural integrity of nuclear paraspeckles.. Proc Natl Acad Sci U S A.

[30] Yoo SM, Ahn AK, Seo T, Hong HB, Chung MA (2008). Centrifugal enhancement of Kaposi's sarcoma-associated virus infection of human endothelial cells in vitro.. J Virol Methods.

[31] Sun R, Lin SF, Gradoville L, Yuan Y, Zhu F (1998). A viral gene that activates lytic cycle expression of Kaposi's sarcoma-associated herpesvirus.. Proc Natl Acad Sci U S A.

[32] Staudt MR, Dittmer DP (2007). The Rta/Orf50 transactivator proteins of the gamma-herpesviridae.. Curr Top Microbiol Immunol.

[33] Lukac DM, Renne R, Kirshner JR, Ganem D (1998). Reactivation of Kaposi's sarcoma-associated herpesvirus infection from latency by expression of the ORF 50 transactivator, a homolog of the EBV R protein.. Virology.

[34] Conrad NK (2008). Chapter 15. Co-immunoprecipitation techniques for assessing RNA-protein interactions in vivo.. Methods Enzymol.

[35] Glaunsinger B, Chavez L, Ganem D (2005). The exonuclease and host shutoff functions of the SOX protein of Kaposi's sarcoma-associated herpesvirus are genetically separable.. J Virol.

[36] Covarrubias S, Richner JM, Clyde K, Lee YJ, Glaunsinger BA (2009). Host shutoff is a conserved phenotype of gammaherpesvirus infection and is orchestrated exclusively from the cytoplasm.. J Virol.

[37] Kumar GR, Shum L, Glaunsinger BA (2011). Importin α-mediated nuclear import of cytoplasmic poly(A) binding protein occurs as a direct consequence of cytoplasmic mRNA depletion.. Mol Cell Biol.

[38] Wilusz JE, Freier SM, Spector DL (2008). 3′ end processing of a long nuclear-retained noncoding RNA yields a tRNA-like cytoplasmic RNA.. Cell.

[39] Sunwoo H, Dinger ME, Wilusz JE, Amaral PP, Mattick JS (2009). MEN epsilon/beta nuclear-retained non-coding RNAs are up-regulated upon muscle differentiation and are essential components of paraspeckles.. Genome Res.

[40] Wang HW, Sharp TV, Koumi A, Koentges G, Boshoff C (2002). Characterization of an anti-apoptotic glycoprotein encoded by Kaposi's sarcoma-associated herpesvirus which resembles a spliced variant of human survivin.. EMBO J.

[41] Vieira J, O'Hearn PM (2004). Use of the red fluorescent protein as a marker of Kaposi's sarcoma-associated herpesvirus lytic gene expression.. Virology.

[42] Myoung J, Ganem D (2011). Generation of a doxycycline-inducible KSHV producer cell line of endothelial origin: Maintenance of tight latency with efficient reactivation upon induction.. J Virol Methods.

[43] Kedes DH, Ganem D (1997). Sensitivity of Kaposi's sarcoma-associated herpesvirus replication to antiviral drugs. Implications for potential therapy.. J Clin Invest.

[44] Faulds D, Heel RC (1990). Ganciclovir. A review of its antiviral activity, pharmacokinetic properties and therapeutic efficacy in cytomegalovirus infections.. Drugs.

[45] Holland LE, Anderson KP, Shipman C, Wagner EK (1980). Viral DNA synthesis is required for the efficient expression of specific herpes simplex virus type 1 mRNA species.. Virology.

[46] Powell KL, Purifoy DJ, Courtney RJ (1975). The synthesis of herpes simplex virus proteins in the absence of virus DNA synthesis.. Biochem Biophys Res Commun.

[47] Jones PC, Roizman B (1979). Regulation of herpesvirus macromolecular synthesis. VIII. The transcription program consists of three phases during which both extent of transcription and accumulation of RNA in the cytoplasm are regulated.. J Virol.

[48] Zhang YJ, Bonaparte RS, Patel D, Stein DA, Iversen PL (2008). Blockade of viral interleukin-6 expression of Kaposi's sarcoma-associated herpesvirus.. Mol Cancer Ther.

[49] Gregory SM, West JA, Dillon PJ, Hilscher C, Dittmer DP (2009). Toll-like receptor signaling controls reactivation of KSHV from latency.. Proc Natl Acad Sci U S A.

[50] Gorlach M, Burd CG, Dreyfuss G (1994). The mRNA poly(A)-binding protein: localization, abundance, and RNA-binding specificity.. Exp Cell Res.

[51] Rowe M, Glaunsinger B, van Leeuwen D, Zuo J, Sweetman D (2007). Host shutoff during productive Epstein-Barr virus infection is mediated by BGLF5 and may contribute to immune evasion.. Proc Natl Acad Sci U S A.

[52] Boyne JR, Jackson BR, Whitehouse A (2010). ORF57: Master regulator of KSHV mRNA biogenesis.. Cell Cycle.

[53] Boyne JR, Colgan KJ, Whitehouse A (2008). Recruitment of the complete hTREX complex is required for Kaposi's sarcoma-associated herpesvirus intronless mRNA nuclear export and virus replication.. PLoS Pathog.

[54] Sahin BB, Patel D, Conrad NK (2010). Kaposi's sarcoma-associated herpesvirus ORF57 protein binds and protects a nuclear noncoding RNA from cellular RNA decay pathways.. PLoS Pathog.

[55] Joachims M, Van Breugel PC, Lloyd RE (1999). Cleavage of poly(A)-binding protein by enterovirus proteases concurrent with inhibition of translation in vitro.. J Virol.

[56] Kerekatte V, Keiper BD, Badorff C, Cai A, Knowlton KU (1999). Cleavage of Poly(A)-binding protein by coxsackievirus 2A protease in vitro and in vivo: another mechanism for host protein synthesis shutoff?. J Virol.

[57] Kuyumcu-Martinez M, Belliot G, Sosnovtsev SV, Chang KO, Green KY (2004). Calicivirus 3C-like proteinase inhibits cellular translation by cleavage of poly(A)-binding protein.. J Virol.

[58] Piron M, Vende P, Cohen J, Poncet D (1998). Rotavirus RNA-binding protein NSP3 interacts with eIF4GI and evicts the poly(A) binding protein from eIF4F.. EMBO J.

[59] Harb M, Becker MM, Vitour D, Baron CH, Vende P (2008). Nuclear localization of cytoplasmic poly(A)-binding protein (PABP-C1) upon rotavirus infection involves interaction of NSP3 with eIF4G and RoXaN.. J Virol.

[60] Montero H, Rojas M, Arias CF, Lopez S (2008). Rotavirus infection induces the phosphorylation of eIF2alpha but prevents the formation of stress granules.. J Virol.

[61] Blakqori G, van Knippenberg I, Elliott RM (2009). Bunyamwera orthobunyavirus S-segment untranslated regions mediate poly(A) tail-independent translation.. J Virol.

[62] Chen Z, Li Y, Krug RM (1999). Influenza A virus NS1 protein targets poly(A)-binding protein II of the cellular 3′-end processing machinery.. EMBO J.

[63] Huang Y, Carmichael GG (1996). Role of polyadenylation in nucleocytoplasmic transport of mRNA.. Mol Cell Biol.

[64] Fortes P, Beloso A, Ortin J (1994). Influenza virus NS1 protein inhibits pre-mRNA splicing and blocks mRNA nucleocytoplasmic transport.. EMBO J.

[65] Alexander L, Denekamp L, Knapp A, Auerbach MR, Damania B (2000). The primary sequence of rhesus monkey rhadinovirus isolate 26-95: sequence similarities to Kaposi's sarcoma-associated herpesvirus and rhesus monkey rhadinovirus isolate 17577.. J Virol.

[66] Dittmer DP, Gonzalez CM, Vahrson W, DeWire SM, Hines-Boykin R (2005). Whole-genome transcription profiling of rhesus monkey rhadinovirus.. J Virol.

[67] Schneider RJ, Weinberger C, Shenk T (1984). Adenovirus VAI RNA facilitates the initiation of translation in virus-infected cells.. Cell.

[68] Lu S, Cullen BR (2004). Adenovirus VA1 noncoding RNA can inhibit small interfering RNA and MicroRNA biogenesis.. J Virol.

[69] Afonina E, Stauber R, Pavlakis GN (1998). The human poly(A)-binding protein 1 shuttles between the nucleus and the cytoplasm.. J Biol Chem.

[70] Brune C, Munchel SE, Fischer N, Podtelejnikov AV, Weis K (2005). Yeast poly(A)-binding protein Pab1 shuttles between the nucleus and the cytoplasm and functions in mRNA export.. RNA.

[71] Chekanova JA, Belostotsky DA (2003). Evidence that poly(A) binding protein has an evolutionarily conserved function in facilitating mRNA biogenesis and export.. RNA.

[72] Hosoda N, Lejeune F, Maquat LE (2006). Evidence that poly(A) binding protein C1 binds nuclear pre-mRNA poly(A) tails.. Mol Cell Biol.

[73] Thakurta AG, Ho Yoon J, Dhar R (2002). Schizosaccharomyces pombe spPABP, a homologue of Saccharomyces cerevisiae Pab1p, is a non-essential, shuttling protein that facilitates mRNA export.. Yeast.

[74] Meyer S, Urbanke C, Wahle E (2002). Equilibrium studies on the association of the nuclear poly(A) binding protein with poly(A) of different lengths.. Biochemistry.

[75] Hilleren P, Parker R (2001). Defects in the mRNA export factors Rat7p, Gle1p, Mex67p, and Rat8p cause hyperadenylation during 3′-end formation of nascent transcripts.. RNA.

[76] Jensen TH, Patricio K, McCarthy T, Rosbash M (2001). A block to mRNA nuclear export in S. cerevisiae leads to hyperadenylation of transcripts that accumulate at the site of transcription.. Mol Cell.

[77] Wyers F, Rougemaille M, Badis G, Rousselle JC, Dufour ME (2005). Cryptic pol II transcripts are degraded by a nuclear quality control pathway involving a new poly(A) polymerase.. Cell.

