# Supplementary figures and images for: A Viral Nuclear Noncoding RNA Binds Re-localized Poly(A) Binding Protein and Is Required for Late KSHV Gene Expression
Source: PLoS Pathog. 2011 Oct 13;7(10):e1002300. doi: 10.1371/journal.ppat.1002300 (PMC3192849; doi:10.1371/journal.ppat.1002300)

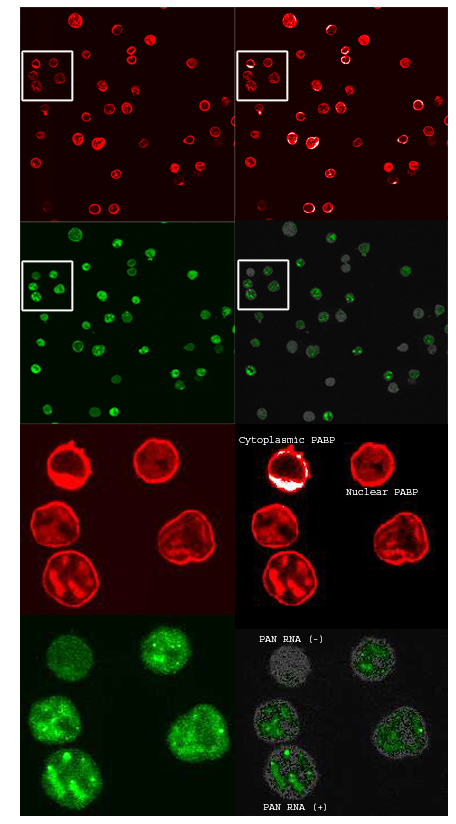

Supplement: Figure S4 — Manual scoring of images taken with a laser scanning confocal microscope indicates that PAN RNA expression and PABPC1 re-localization are highly correlated. Left panels show wide field images of PABPC1 (top, red) and PAN RNA (bottom, green). Right panels show these same images, except that saturated pixels are identified in white and background pixels are in gray. Saturated and background signals aided scoring since cytoplasmic PABPC1 stained very intensely compared to re-localized PABPC1 and since the anti-PAN RNA oligonucleotide probes described in Materials and Methods sometimes gave significant background. Images of representative regions in the lower 4 panels are indicated by white boxes in the upper 4 panels. A total of 450 cells were scored independently by two individuals. The percent of cells with re-localized PABPC1 that also expressed PAN RNA was scored as 73% by person 1 (larger sample size), and 90% by person 2 (smaller sample size), for an average score was 76%. (TIF) [file ppat.1002300.s004.tif]

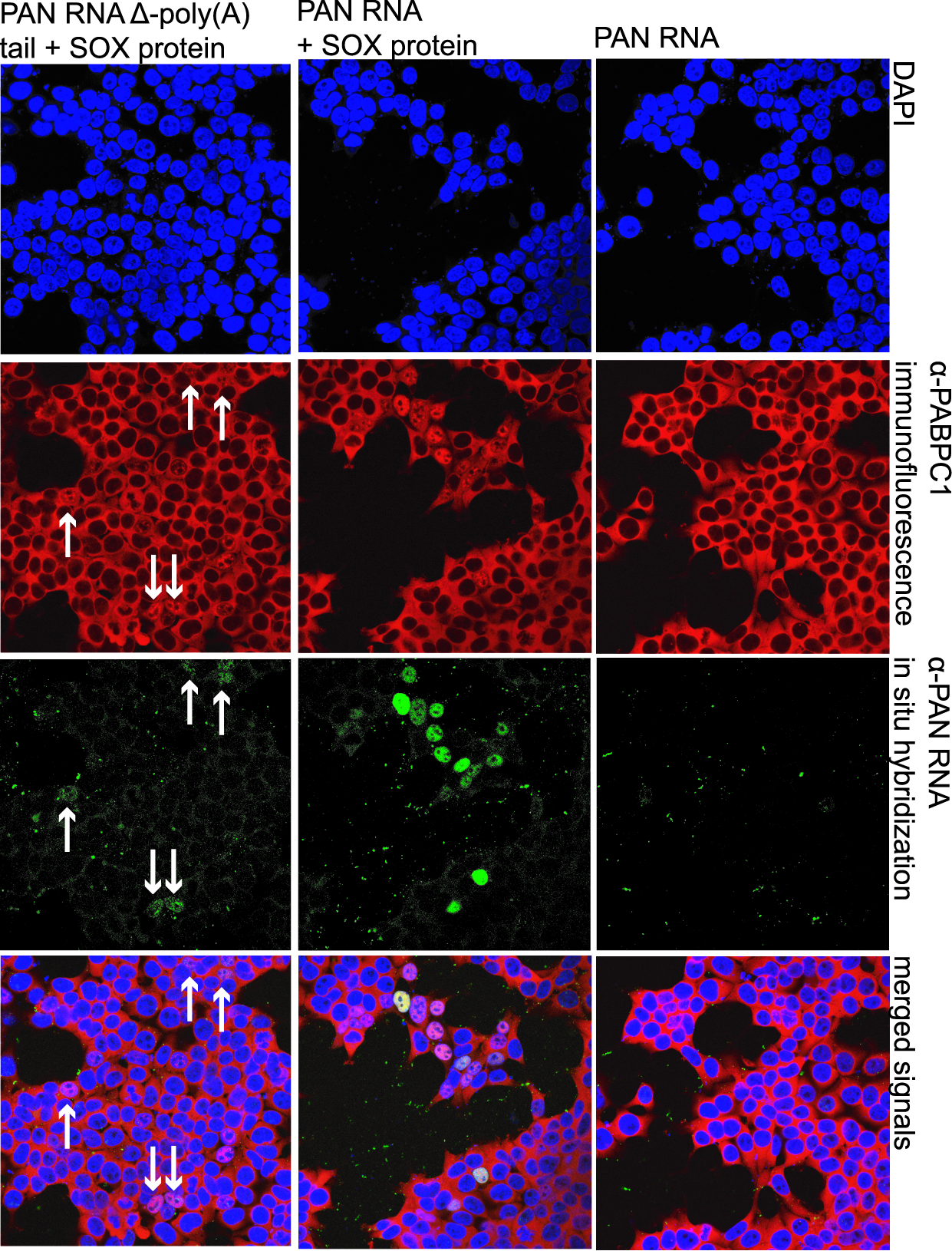

Supplement: Figure S5 — Replacing the poly(A) signal of PAN RNA with a U7 snRNP-dependent 3′-end formation signal (PAN RNA Δ-poly(A) tail) [6] reduces the effect of SOX co-transfection on PAN accumulation in 293T cells, as viewed by confocal microscopy. Note that some cells in which PABPC1 appears nuclear show slightly enhanced signal even for PAN RNA lacking a poly(A) tail, as indicated with white arrows, consistent with the northern blot results (see Fig. 2A). (TIF) [file ppat.1002300.s005.tif]
